# Hepatocyte Growth Hormone Receptor Ablation Is Associated With Aging Phenotypes and Hepatic Mitochondrial Dysfunction

**DOI:** 10.1111/acel.70741

**Published:** 2026-09-25

**Authors:** Kangkang Yang, Yuli Jian, Fusheng Pang, Ming Ying, Qian Yang, Nian Liu, Shujing Wang, Yingjie Wu

**Affiliations:** ^1^ Liaoning Provence Key Lab of Genome Engineered Animal Models, Institute of Genome Engineered Animal Models for Human Diseases, National Center of Genetically Engineered Animal Models for International Research Dalian Medical University Dalian Liaoning P.R. China; ^2^ Liaoning Provincial Core Lab of Glycobiology and Glycoengineering, Department of Biochemistry and Molecular Biology, College of Basic Medical Sciences Dalian Medical University Dalian Liaoning P.R. China; ^3^ Key Laboratory of Endocrine Glucose & Lipids Metabolism and Brain Aging, Ministry of Education Shandong Provincial Hospital Affiliated to Shandong First Medical University & Shandong Academy of Medical Sciences Jinan Shandong P.R. China

**Keywords:** age‐related pathologies, aging, ectopic lipid accumulation, GH‐GHR, mitochondrial dysfunction

## Abstract

Aging drives physiological decline and predisposes individuals to multiple age‐related pathologies, constituting a major global health challenge. Growth hormone receptor (GHR), a critical regulator of growth, development, and metabolism, has emerged as a potential therapeutic target. However, its precise role in aging and age‐related diseases remains incompletely defined. Here, we demonstrate that hepatocyte‐specific GHR knockout mice display accelerated aging‐related phenotypes, characterized by shortened lifespan, enhanced cellular senescence, reduced metabolic stress resilience, cognitive decline, impaired bone mineralization, and exacerbated inflammaging. Elevated circulating GH following hepatocyte‐specific GHR ablation mediates adipose‐liver crosstalk that promotes adipose lipolysis and CD36‐dependent hepatic steatosis. Hepatocyte‐specific GHR deficiency also promotes liver aging and aggravates age‐related hepatic pathologies in both naturally aged and high‐fat diet (HFD)‐fed mice. Mechanistically, loss of hepatic GHR impairs STAT5b phosphorylation while upregulating PPARγ expression. Enhanced nuclear translocation of PPARγ activates transcription of *Pdk4* and *Cd36*, leading to mitochondrial damage and ectopic lipid accumulation. Together, dysregulated lipid metabolism and mitochondrial dysfunction establish a self‐amplifying vicious cycle that accelerates aging and its pathophysiology. Pharmacological inhibition of PDK4 in vivo effectively ameliorates the age‐related pathologies induced by hepatocyte‐specific GHR ablation. These findings identify hepatic GHR signaling as an important contributor to age‐related hepatic pathology and a candidate node within the broader network of factors driving systemic aging phenotypes, highlighting hepatocyte GHR signaling as a promising therapeutic target for age‐related liver disorders.

## Graphical abstract

Hepatocyte‐specific GHR ablation is associated with hepatic and age‐related pathologies, triggering adipose‐liver crosstalk via elevated circulating GH and subsequent CD36‐dependent hepatic steatosis. This loss impairs STAT5b phosphorylation and enhances PPARγ nuclear activation, upregulating *Pdk4* and *Cd36* to drive mitochondrial dysfunction and ectopic lipid accumulation, a vicious cycle reversible by targeting PDK4.

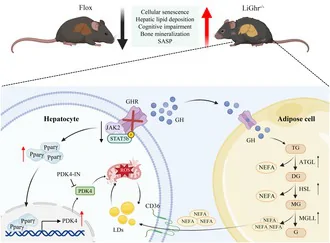

## 1 Introduction

Aging represents a complicated and irreversible biological process, characterized by a gradual decline in physiological functions and an elevated risk of age‐related diseases, posing a persistent global public health challenge (Dujon et al. 2026). Approximately two‐thirds of daily deaths worldwide are attributable to aging‐associated conditions, a burden particularly evident in industrialized nations (Chang et al. 2019). Mechanistically, aging is driven by dysregulation of key biological processes, including increased senescence‐associated β‐galactosidase (SA‐β‐gal) activity, cell cycle arrest mediated by p16^INK4a^ (p16, encoded by *CDKN2A*) and p21^CIP1^ (p21, encoded by *CDKN1A*), mitochondrial dysfunction, reduced antioxidant capacity, activation of the senescence‐associated secretory phenotype (SASP), and impaired lipid metabolism (Yamauchi et al. 2024; Zeng et al. 2024). These alterations not only accelerate aging but also underlie the development of diverse pathologies such as kidney disease, liver fibrosis, cardiovascular disorders, and metabolic syndrome (Du et al. 2025; Duan et al. 2023; Liu et al. 2024; Sun et al. 2025). Collectively, they reduce quality of life in older adults, increase healthcare costs, and impose substantial socioeconomic burdens. Consequently, contemporary aging research emphasizes extending healthspan rather than merely prolonging lifespan. With the rapid demographic shift toward aging populations, there is an urgent need to address age‐related morbidities through mechanistic insight and targeted intervention.

Growth hormone (GH), secreted by the anterior pituitary, exerts critical regulatory effects on mammalian growth, development, and metabolism via its transmembrane receptor, GHR (Poudel et al. 2025). Dysregulation of GH signaling results in disorders ranging from GH deficiency to acromegaly, while aberrant GH‐GHR activity contributes to oncogenic transformation in multiple tissues (Basu et al. 2025). Emerging evidence highlights the role of GHR in modulating aging. For instance, global GHR knockout (GHR^−/−^) mice display severe postnatal growth retardation but also exceptional longevity, representing the longest‐lived laboratory murine strain (Coschigano et al. 2003). These mice exhibit enhanced insulin sensitivity, improved glucose homeostasis, and resistance to obesity‐associated adipose inflammation (Dominici et al. 2000; Young et al. 2020). Adipose tissue is recognized as a central regulator of systemic aging, showing some of the earliest and most pronounced senescence‐associated changes (Yu et al. 2024). Indeed, adipocyte‐specific GHR deletion (AdGHR^−/−^) modestly extends lifespan in both sexes and improves frailty scores and grip strength in aged mice, despite increased adiposity and reduced insulin levels (List et al. 2022). AdGHR^−/−^ mice exacerbate HFD‐fed obesity via enhanced lipogenesis and adipocyte differentiation, yet simultaneously confer hepatic protection against ectopic lipid deposition, establishing a “healthy obesity” phenotype (Ran et al. 2019). Our latest work further demonstrated that aged AdGHR^−/−^ mice exhibited extended healthspan, markedly improved cognitive function, reduced age‐related lipid redistribution, restored glucose homeostasis, and maintained a low‐inflammatory state (Ma et al. 2026; Zhang et al. 2026). In contrast, hepatic aging disrupts systemic metabolic homeostasis, increasing susceptibility to metabolic disorders (Kundu et al. 2023). Hepatocellular senescence also drives multi‐organ senescence and dysfunction (Kiourtis et al. 2024). Targeted deletion of hepatic GHR induces lipogenic transcriptional programs, leading to de novo lipogenesis and pathological lipid deposition within hepatic tissues (Fan et al. 2009; Vázquez‐Borrego et al. 2023; Yu et al. 2026). However, the precise role of hepatic GHR in aging remains poorly understood.

In this study, hepatocyte‐specific GHR knockout (LiGHR^−/−^) mice were generated to explore the contribution of hepatic GHR to aging‐related phenotypes. We found that male LiGHR^−/−^ mice exhibited accelerated aging‐related phenotypes, including shortened lifespan, impaired metabolic stress resilience, cognitive decline, reduced bone mineralization, and systemic inflammation, although this model does not permit dissociation of liver‐intrinsic effects from those mediated by elevated circulating GH acting on GHR‐intact peripheral tissues. Hepatocyte‐specific GHR ablation also exacerbated liver aging in both naturally aged and HFD‐fed mice, as indicated by elevated senescence markers, fibrotic remodeling, heightened SASP, and ectopic lipid deposition. Mechanistically, loss of hepatic GHR reduced STAT5b phosphorylation while upregulating PPARγ expression. Nuclear translocation of PPARγ activated *Cd36* and *Pdk4*, driving ectopic lipid accumulation and mitochondrial dysfunction, thereby contributing to systemic aging phenotypes. Together, these findings identify hepatic GHR signaling as an important contributor to hepatic aging and a promising therapeutic target for mitigating age‐related liver pathogenesis.

## 2 Methods

### 2.1 Antibodies

The following antibodies were used in this study: Proteintech (China): anti‐F4/80 (29414‐1‐AP), anti‐HSL (17333‐1‐AP), anti‐ATGL (55190‐1‐AP), anti‐MGLL (14986‐1‐AP), anti‐CD36 (18836‐1‐AP), anti‐p53 (60283‐2‐Ig), anti‐PDK4 (12949‐1‐AP), anti‐α‐SMA (14395‐1‐AP), anti‐PPARγ (16643‐1‐AP), anti‐Beclin1 (11306‐1‐AP), anti‐phospho‐mTOR (Ser2448, 80596‐1‐RR), anti‐mTOR (66888‐1‐Ig), anti‐SIRT5 (15122‐1‐AP), anti‐NF‐κB p65 (80979‐1‐RR), anti‐phospho‐NF‐κB p65 (Ser468, 82335‐1‐RR), and anti‐LC3B (14600‐1‐AP). Thermo Fisher Scientific (USA): anti‐p16 (MA5‐17142) and anti‐p62/SQSTM1 (PA5‐20839). ABclonal (China): anti‐phospho‐Histone H2AX‐S139 (γH2AX, AP0687), anti‐STAT5B (A21613), anti‐SIRT1 (A11267SP), and anti‐p21 (A19094). Affinity Biosciences (China): anti‐IL1β (#AF5103), anti‐IL6 (#DF6087), anti‐phospho‐STAT5B (Ser731, #AF3340), and anti‐Col1a1 (#AF7001). Abbkine (China): anti‐phospho‐AMPKα1/2 (Thr183/172, ABP0052) and anti‐AMPKα1/2 (ABP0060). Servicebio (China): anti‐β‐Actin (GB15003‐100), anti‐GAPDH (GB11002‐100), and anti‐β‐Tubulin (GB15140‐100).

### 2.2 Generation of Hepatocyte‐Specific GHR Knockout Mice and Mice Experiments

All mice were maintained in the specific pathogen‐free animal facility of Dalian Medical University (Dalian, China) under controlled conditions (20°C–22°C, 12‐h light/dark cycle) with *ad libitum* access to food and water. Heterozygous mice were generated by crossing Flox mice (Wu et al. 2009) with Albumin‐Cre transgenic mice (Jackson Laboratory). LiGHR^−/−^ mice were obtained by further breeding of heterozygous animals. Genotyping was performed by polymerase chain reaction (PCR) using primers listed in Table S1. Mice were fed either a regular chow (RC) or HFD. The 20‐month‐old LiGHR^−/−^ and Flox mice were treated once weekly for 16 weeks with either vehicle or the PDK4‐specific inhibitor (PDK4‐IN, 5 mg/kg (Low) or 10 mg/kg (High), intraperitoneal injection (i.p.)) to determine whether PDK4 inhibition could delay or ameliorate the age‐related phenotypes induced by hepatocyte‐specific GHR ablation. All animal experiments were conducted in accordance with the guidelines of the Institutional Animal Care and Use Committee (IACUC) of Dalian Medical University (Approval No. L251029543).

### 2.3 Behavioral Experiments in Mice

The Morris water maze (MWM) test was conducted as previously described (Vorhees and Williams 2006). Any‐maze software (Global Biotech Inc., China) was used to quantify and analyze several parameters, including the frequency of platform crossings, the duration spent in the target quadrant, and the swimming trajectory of the mice. For the Y‐maze test, following a 5‐min acclimation to the apparatus, each mouse was subjected to a 3‐min free exploration session while its movement trajectory was monitored. The maze was cleaned meticulously with 70% ethanol followed by distilled water, and a 5‐min interval was maintained prior to the commencement of the next trial. We performed the open‐field test by placing mice individually into a quiet, opaque chamber (50 × 50 × 40 cm) for a 5‐min free exploration. Trajectories and time spent in the central zone were recorded to assess locomotor activity. The testing environment was kept quiet, and the chamber was wiped with 75% ethanol between trials.

### 2.4 Micro‐CT

Right femurs were isolated from each mouse, fixed in 4% paraformaldehyde overnight, and dehydrated in 70% ethanol for 48 h. Bone microarchitecture was then assessed using micro‐CT (SkyScan 1276, Bruker) at 10 μm resolution, 70 kV, and 140 μA.

### 2.5 Transmission Electron Microscope (TEM) and Images Analysis

Following fixation and dehydration, liver tissue samples were prepared and contrasted with uranyl acetate and lead citrate. Ultrathin sections were then assessed under a JEM‐2000EX microscope (JEOL, Japan) for imaging.

### 2.6 Glucose Tolerance Test (GTT) and Insulin Tolerance Test (ITT)

For the GTT, mice were fasted overnight, and blood glucose levels were measured using a glucose meter (Roche, Mannheim, Germany) at 0, 15, 30, 60, and 120 min after intraperitoneal injection of glucose (2 g/kg). For the ITT, mice were fasted for 6 h, and blood glucose levels were measured at 0, 15, 30, and 60 min following intraperitoneal injection of insulin (0.75 IU/kg, Jiangsu Wanbang Biochemistry Medicine Co., China).

### 2.7 RNA Sequencing (RNA‐Seq)

Following the supplier's instructions, the RNA was diluted in buffer and processed through reverse transcription, pre‐amplification, cDNA purification, and construction of circular single‐stranded DNA libraries. Differentially expressed genes (DEGs) were identified using the DESeq2 criteria of |Log_2_ (Fold change)| > 1 and *p*‐value < 0.05. KEGG pathway enrichment analysis was performed using the clusterProfiler package in R.

### 2.8 Establishment of GHR‐Knockdown Cells and Free Fatty Acids (FFA) Treatment

Short hairpin RNAs (shRNAs) targeting GHR were constructed and synthesized by GenePharma, including shGHR‐1: 5′‐CCC CAG TTC TGC AAA GAA TCA‐3′, shGHR‐2: 5′‐GGC CCT ATA TGG TTA ACA TAC‐3′, and shGHR‐3: 5′‐CTC AGT CAC AGA AGT TAA ATA‐3′. Lentiviruses were transfected into AML12 cells following the manufacturer's instructions, and stable cell lines were obtained via puromycin selection (Beyotime Biotechnology, China).

To induce cellular senescence, oleate acid (OA, O7501, Sigma‐Aldrich) and sodium palmitate (PA, P9767, Sigma‐Aldrich) were prepared as FFA according to a previously described method (Gao et al. 2020). Briefly, bovine serum albumin (BSA, 0.5 M, 9048‐46‐8, Sigma‐Aldrich, Germany) was dissolved in 150 mM NaCl, OA and PA were subsequently dissolved in the BSA solution to generate a 5 mM FFA mixture (oleate: palmitate molar ratio = 2:1), which was filtered through a 0.22 μm membrane. The stock solution was diluted 1:10 in culture medium to a final concentration of 500 μM. Cells were treated with FFA for 7 days, with the medium refreshed daily. To explore whether GH directly regulates CD36 and PDK4 expression, control and shGHR cells were treated with FFA for 48 h and with GH (500 ng/mL) for 1 h (Kim et al. 2012).

### 2.9 Generation of STAT5b‐Overexpressing and PPARγ‐Knockdown AML12 Cells

To generate STAT5b‐overexpressing (STAT5b‐OE) AML12 cells, recombinant pcDNA3.1‐STAT5b and empty vector control were constructed by GenePharma (Shanghai, China). AML12 cells were transfected with the plasmids using Lipofectamine 3000 (Invitrogen, USA) according to the manufacturer's instructions.

For PPARγ knockdown, three small interfering RNA (siRNA) targeting mouse Pparγ were synthesized by GenePharma (Shanghai, China): si‐PPARγ‐1 (sense: 5′‐GCAAGAGAUCACAGAGUAUTT‐3′, antisense: 5′‐AUACUCUGUGAUCUCUUGCTT‐3′), si‐PPARγ‐2 (sense: 5′‐GGCCUCCCUGAUGAAUAAATT‐3′, antisense: 5′‐UUUAUUCAUCAGGGAGGCCTT‐3′), si‐PPARγ‐3 (sense: 5′‐GGAGCCUAAGUUUGAGUUUTT‐3′, antisense: 5′‐AAACUCAAACUUAGG CUCCTT‐3′). A non‐targeting siRNA was used as a negative control (si‐control; sense: 5′‐UUCUCCGAACGUGUCACGUTT‐3′, antisense: 5′‐ACGUGACACGUUCGGAGAATT‐3′). AML12 cells were transiently transfected with siRNAs using Lipofectamine 3000 according to the manufacturer's protocol. Cells were harvested 48 h after transfection for subsequent analyses.

### 2.10 Dual‐Luciferase Reporter Gene Assay

The PDK4 wild type (WT) promoter and PDK4 mutant (MUT) promoter were cloned into the pGL4‐Basic vector (GenePharma, China). Lipofectamine 3000 (Invitrogen, USA) was used to co‐transfect HEK293T cells with PDK4‐WT or PDK4‐MUT (GenePharma, China) and pRL‐TK Renilla plasmid (GenePharma, China) with pcDNA3.1 or pcDNA3.1‐PPARγ (GenePharma, China). After 48 h of transfection, the dual‐luciferase reporter assay was conducted using a Dual Luciferase Reporter Assay Kit (KTA8010, Abbkine, China).

### 2.11 Oxygen Consumption Rate (OCR) Assay

Cells from each group were seeded onto XFe‐48 microplates at a density of 1.0 × 10^5^ cells per well and cultured overnight. Oligomycin (1.5 μM), FCCP (1.0 μM), and rotenone/antimycin A (0.5 μM) were sequentially injected, and key parameters, including non‐mitochondrial oxygen consumption, basal respiration, maximal respiration, ATP production, and spare respiratory capacity, were analyzed using Wave software (Agilent).

### 2.12 Mito‐Tracker and BODIPY Staining

After reaching about 70% confluence in confocal dishes, cells were stained with Mito‐Tracker (1 mM, C1997S, Beyotime Biotechnology, China) or BODIPY 493/503 (1 μg/mL, C2053S, Beyotime Biotechnology, China) for 30 min, followed by nuclear staining with DAPI (P0131, Beyotime Biotechnology, China). Fluorescence images were captured.

### 2.13 SA‐β‐Gal Staining

Following fixation and PBS washing, cells or tissues were stained for SA‐β‐Gal activity using a designated kit (Beyotime Biotechnology, China) with subsequent incubation at 37°C for 12 to 24 h, and finally examined under a Nikon light microscope (Japan).

### 2.14 Histological Analysis

Liver injury and fibrosis were further evaluated using PAS (G1280, Solarbio, China) and Masson's trichrome (C0189S, Beyotime Biotechnology, China) staining according to the manufacturer's protocols. For immunohistochemistry (IHC), paraffin sections were deparaffinized, hydrated, blocked, and incubated with primary antibodies, followed by secondary antibodies, and developed using a DAB kit (ZLI‐9017, ZSGB‐Bio, China). All stained sections were analyzed under a light microscope (Nikon, Japan).

### 2.15 Real‐Time Quantitative PCR (RT‐qPCR)

Total RNA from cultured cells or tissues was extracted using RNAiso Plus reagent (Takara, Japan) following the manufacturer's protocols and reverse‐transcribed into cDNA. RT‐qPCR was performed using SYBR Green Master Mix (TransGen Biotech, China) in a 7900HT Fast Real‐Time PCR System (Applied Biosystem, USA). Gene expression was quantified by the 2^−ΔΔCt^ method and normalized to β‐Actin. Primers sequences are listed in Table S2.

### 2.16 ELISA Kit Assays

Proinflammatory cytokine levels were measured using mouse IL1β (E‐EL‐M0037), TNFα (E‐EL‐M3063), and IL6 (E‐EL‐M0044) ELISA kit (Elabscience, China) following the manufacturer's instructions. Additionally, levels of TG, T‐CHO, GH, NEFA, LDL‐C, HDL‐C, AST, ALT, SOD, CAT, GSH‐Px, and IGF1 were quantified by sandwich ELISA kits according to the corresponding manufacturers' protocols (Nanjing Jiancheng Bioengineering Institute, China).

### 2.17 Immunofluorescence

After deparaffinization, hydration, and blocking, liver and adipose tissue sections were incubated with primary antibodies (F4/80 (1:200), CD36 (1:250), α‐SMA (1:500), and UCP1 (1:500)) for 24 h at 4°C, followed by incubation with the corresponding fluorescent secondary antibodies. Nuclei were stained with DAPI (P0131, Beyotime Biotechnology, China), and sections were imaged using a fluorescence microscope (Nikon, Japan).

### 2.18 Western Blot and Co‐Immunoprecipitation (Co‐IP)

Tissue specimens and cultured cells were harvested and lysed for protein extraction. Selected tissues were further fractionated into nuclear and non‐nuclear fractions. Equal protein loading was resolved by SDS‐PAGE and electroblotted onto nitrocellulose membranes (Millipore, USA). Subsequent to blocking with 5% skimmed milk or BSA, membranes were probed with specific primary antibodies and corresponding HRP‐linked secondary antibodies. Immunoreactive bands were visualized with an ECL system.

For Co‐IP, protein lysates (1 mg) were subjected to an overnight incubation at 4°C with gentle rotation in the presence of the indicated primary antibody (5 μL). After washing, 35 μL of protein A + G‐conjugated beads (Beyotime Biotechnology, China) were added and rotated at 4°C for 4 h. Bound proteins were eluted by boiling in 2× loading buffer and subsequently analyzed by Western blot.

### 2.19 Statistical Analysis

Data are presented as mean ± standard error of the mean (SEM). Statistical analyses were performed using GraphPad Prism (Version 9.0). Two‐tailed Student's *t*‐tests were used to compare differences between two groups, while one‐way or two‐way ANOVA was applied for comparisons among multiple groups. Differences were considered statistically significant at *p* < 0.05.

## 3 Results

### 3.1 Ablation of Hepatocyte‐Specific GHR Exacerbates Age‐Associated Pathologies

**FIGURE 1:**
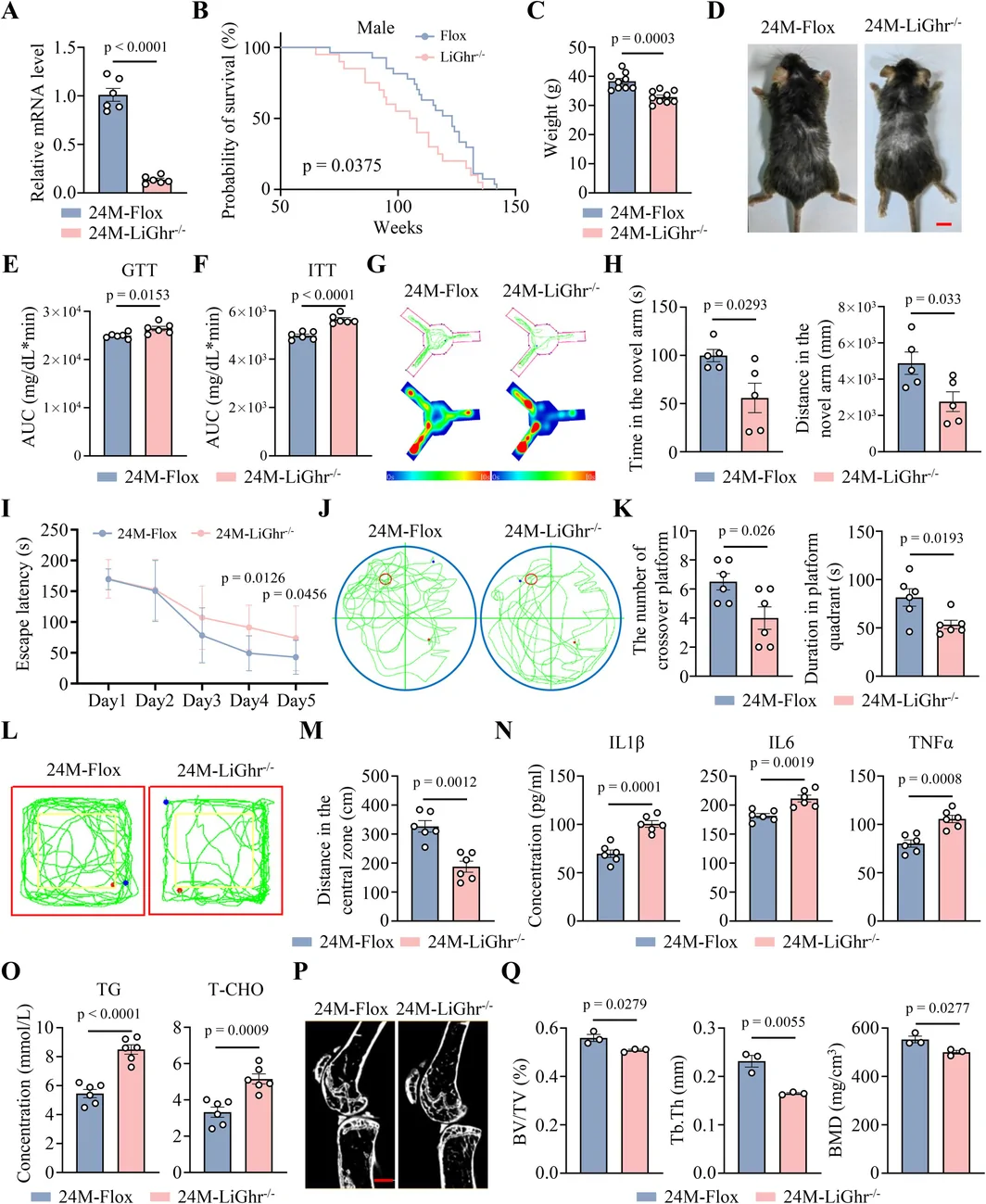
Hepatocyte‐specific deletion of GHR accelerates aging, inflammation, musculoskeletal frailty, and cognitive impairment. (A) RT‐qPCR analysis of *Ghr* mRNA expression in the livers of 24M‐Flox and 24M‐LiGHR^−/−^ mice, *n* = 6. (B) Survival curves of male Flox (*n* = 27) and LiGHR^−/−^ (*n* = 20) mice. (C) Body weights of male 24M‐Flox and 24M‐LiGHR^−/−^ mice, *n* = 9. (D) Representative images of 24M‐Flox and 24M‐LiGHR^−/−^ mice. (E) Area under the curve (AUC) for GTT in male 24M‐Flox and 24M‐LiGHR^−/−^ mice, *n* = 6. (F) AUC for ITT in male 24M‐Flox and 24M‐LiGHR^−/−^ mice, *n* = 6. (G) Representative images from the Y‐maze test, including behavioral tracking maps (top) and occupancy heatmaps (bottom) for 24M‐Flox and 24M‐LiGHR^−/−^ mice. (H) Time spent and distance traveled in the novel arm of the Y‐maze, *n* = 5. (I) Escape latency during the 5‐day Morris water maze (MWM) training period. (J) Representative behavioral tracking maps during the MWM testing phase. (K) Platform crossings and target quadrant occupancy during the MWM test, *n* = 6. (L) Representative behavioral tracking maps from the open field test. (M) Distance traveled in the central zone during the open field test, *n* = 6. (N) Serum levels of IL1β, IL6, and TNFα were measured by ELISA, *n* = 6. (O) Serum TG and T‐CHO levels, *n* = 6. (P) Representative images of right femurs, *n* = 3, scale bar = 1 mm. (Q) Bone mineral density (BMD), bone volume fraction (BV/TV), and trabecular thickness (Tb.Th) of right femurs.

To elucidate the contribution of hepatic GHR to aging‐related phenotypes, LiGHR^−/−^ mice were generated through crossing GHR^flox/flox^ (Flox) mice with Albumin‐Cre mice (Figure S1A). PCR genotyping confirmed Cre expression exclusively in LiGHR^−/−^ mice (Figure S1B). Analysis of tissue specificity demonstrated that Cre expression was exclusive to the liver and not evident in the examined adipose tissue, heart, spleen, kidney, lung, brain, or small intestine (Figure S1C). Consistently, LiGHR^−/−^ mice exhibited a pronounced decrease in hepatic *Ghr* mRNA expression (Figure 1A).

The survival of Flox and LiGHR^−/−^ mice of both sexes was monitored. Male LiGHR^−/−^ mice exhibited a shortened lifespan, whereas no significant difference was observed in females, indicating a sex‐dependent effect of hepatocyte‐specific GHR knockout (Figures 1B and S1D). At 24 months (24 M), male LiGHR^−/−^ mice showed significantly lower body weight than controls (Figure 1C). Aging is accompanied by systemic changes, including alterations in physical appearance; notably, 24M‐LiGHR^−/−^ mice exhibited sparser fur, reflecting a pronounced aging phenotype (Figure 1D). Insulin resistance, a hallmark of age‐related metabolic syndrome, was exacerbated in old mice (Yang et al. 2023). GTT and ITT revealed impaired glucose homeostasis and reduced insulin sensitivity in male 24M‐LiGHR^−/−^ mice compared to 24M‐Flox controls (Figures 1E,F and S1E,F), indicating a compromised metabolic response.

Cognitive function was evaluated using behavioral tests. In the Y‐maze, 24M‐LiGHR^−/−^ mice spent less time exploring the novel arm and covered shorter distances than 24M‐Flox mice (Figure 1G,H). In the MWM test, 24M‐LiGHR^−/−^ mice required more time to locate the hidden platform, decreased time in the target quadrant, and reduced number of platform crossings, indicating impaired spatial learning and memory retention (Figure 1I–K). In the open field test, LiGHR^−/−^ mice traveled shorter distances in the central area, further reflecting cognitive decline (Figure 1L,M). Collectively, these results demonstrate that male 24M‐LiGHR^−/−^ mice exhibit deficits in learning and memory, indicative of cognitive impairment.

A persistent, low‐grade inflammatory state is recognized as a fundamental characteristic of aging, often termed “inflammaging” (Wang et al. 2026). Consistent with this, serum levels of proinflammatory cytokines (TNFα, IL6, and IL1β) were elevated in 24M‐LiGHR^−/−^ mice (Figure 1N). The triglyceride (TG) and total cholesterol (T‐CHO) contents in the serum of 24M‐LiGHR^−/−^ mice were also markedly increased (Figure 1O). Considering musculoskeletal fragility as another key feature of aging, femoral bone integrity was assessed by micro‐CT. Male 24M‐LiGHR^−/−^ mice exhibited reduced bone mineral density (BMD), bone volume fraction (BV/TV), and trabecular thickness (Tb.Th) (Figure 1P,Q).

Taken together, these findings demonstrate that hepatocyte‐specific GHR ablation is associated with multiple age‐related phenotypes in vivo, including hair loss, chronic inflammation, metabolic dysregulation, musculoskeletal frailty, and cognitive decline. As these phenotypes involve tissues outside the liver, the relative contributions of hepatic GHR loss versus elevated circulating GH acting on peripheral GHR‐intact tissues cannot be resolved by this model (see Discussion). These findings highlight hepatic GHR as an important node influencing the aging process.

### 3.2 Hepatocyte‐Specific GHR Deletion Aggravates Liver Aging and Age‐Related Liver Pathologies

**FIGURE 2:**
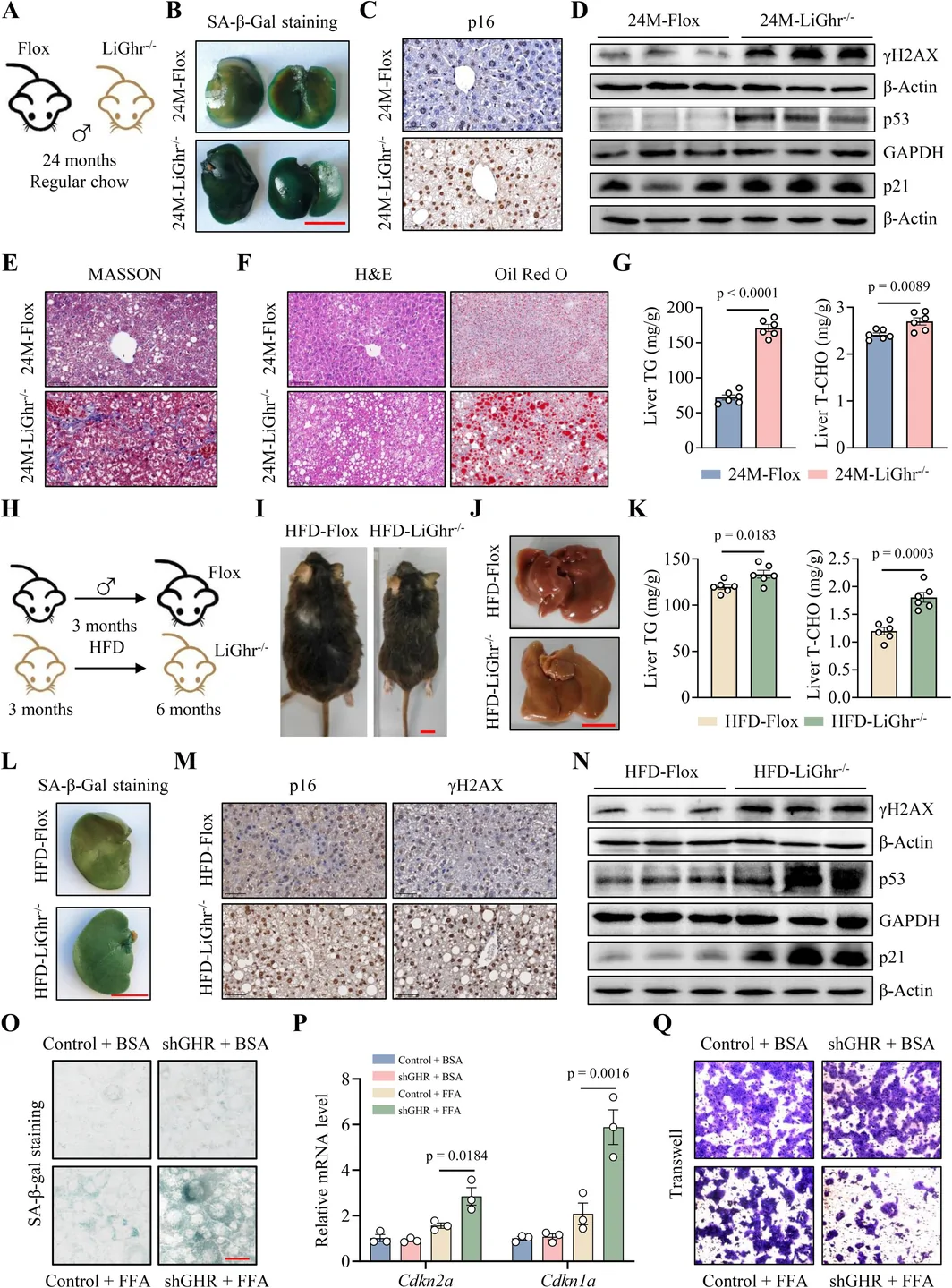
Hepatocyte‐specific GHR deletion promotes hepatic senescence, inflammation, fibrotic remodeling, and ectopic lipid deposition. (A) Schematic illustration of the experimental design. Male Flox and LiGHR^−/−^ mice were fed regular chow for about 24 months, then euthanized for analysis, *n* = 6. (B) Representative SA‐β‐gal staining images of liver tissues from 24M‐Flox and 24M‐LiGHR^−/−^ mice. (C) Immunohistochemical detection of p16 in liver sections of 24M‐Flox and 24M‐LiGHR^−/−^ mice. Scale bar: 50 μm. (D) Western blot analysis of γH2AX, p53, and p21 protein levels in liver tissues from 24M‐Flox and 24M‐LiGHR^−/−^ mice. (E) Representative Masson's trichrome staining of liver sections from 24M‐Flox and 24M‐LiGHR^−/−^ mice. Scale bar: 50 μm. (F) Representative images of H&E and Oil Red O staining of liver sections from 24M‐Flox and 24M‐LiGHR^−/−^ mice. Scale bar: 50 μm. (G) Liver TG and T‐CHO levels in 24‐month‐old male Flox and LiGHR^−/−^ mice. (H) Experimental workflow for HFD‐induced liver aging. Male 3‐month‐old Flox and LiGHR^−/−^ mice were fed HFD for 3 months, then euthanized, *n* = 6. (I) Representative images of male HFD‐Flox and HFD‐LiGHR^−/−^ mice. Scale bar: 1 cm. (J) Representative liver images from HFD‐Flox and HFD‐LiGHR^−/−^ mice. Scale bar: 1 cm. (K) Liver TG and T‐CHO levels in HFD‐Flox and HFD‐LiGHR^−/−^ mice. (L) Representative SA‐β‐gal staining images of liver tissues from HFD‐Flox and HFD‐LiGHR^−/−^ mice. (M) Immunohistochemical detection of p16 and γH2AX in liver sections from HFD‐Flox and HFD‐LiGHR^−/−^ mice. Scale bar: 50 μm. (N) Western blot analysis of γH2AX, p53, and p21 protein levels in liver tissues from HFD‐Flox and HFD‐LiGHR^−/−^ mice. (O) Representative SA‐β‐gal staining images of cells treated with FFA or BSA. Scale bar: 50 μm. (P) RT‐qPCR analysis of *Cdkn1a* and *Cdkn2a* mRNA expression in BSA‐control, BSA‐shGHR, FFA‐control, and FFA‐shGHR cells. (Q) Transwell assay assessing cell migration abilities in the indicated groups.

To investigate the role of hepatic GHR in aging, male 24‐month‐old Flox and LiGHR^−/−^ mice were further analyzed (Figure 2A). The SA‐β‐Gal activity was markedly elevated in multiple organs of 24M‐LiGHR^−/−^ mice (Figures 2B and S2A). Although the absolute liver weights were comparable between groups, the liver‐to‐body weight ratio was increased in LiGHR^−/−^ mice (Figure S2B–D). Consistent with enhanced senescence, hepatic expression of p16, p21, and p53 was upregulated, and γH2AX, a DNA damage marker, was significantly increased, indicating hepatocyte growth arrest and DNA damage accumulation (Figures 2C,D and S2E). Fibrotic marker α‐SMA was significantly elevated in 24M‐LiGHR^−/−^ livers (Figure S2F), and Masson's trichrome staining revealed severe fibrosis and tissue damage (Figure 2E). Inflammatory response was also exacerbated, as evidenced by increased IL6 and IL1β expression (Figure S2G). Histopathological analysis using hematoxylin and eosin (H&E) and Oil Red O staining demonstrated marked hepatic steatosis and lipid accumulation (Figure 2F). Correspondingly, hepatic TG and T‐CHO levels were elevated in LiGHR^−/−^ mice (Figure 2G), highlighting aggravated lipid dysregulation. As male LiGHR^−/−^ mice aged, hepatic lipid accumulation progressively increased (Figure S2H,I), accompanied by elevated expression of senescence markers and inflammatory proteins (Figure S2J,K). These findings suggest that accelerated aging in male LiGHR^−/−^ mice may be associated with age‐dependent ectopic lipid deposition in the liver.

Hepatic steatosis and senescence interact bidirectionally, reinforcing pathological progression (Ogrodnik et al. 2017). To examine whether hepatic GHR deletion exacerbates diet‐induced liver aging, male LiGHR^−/−^ and Flox mice were subjected to a 3‐month HFD challenge (Figure 2H). HFD‐LiGHR^−/−^ mice exhibited reduced weight gain relative to HFD‐Flox mice (Figures 2I and S3A). HFD exposure led to paler and yellower livers with extensive lipid droplet accumulation evidenced by H&E and Oil Red O staining (Figures 2J and S3B), and the liver‐to‐body weight ratio was higher in HFD‐LiGHR^−/−^ mice (Figure S3C). Hepatic TG and T‐CHO contents were increased in HFD‐LiGHR^−/−^ mice (Figure 2K), and serum analyses showed elevated LDL‐C, TG, and T‐CHO levels, indicating severe dyslipidemia (Figure S3D,E). Liver function markers (AST and ALT) were increased, and fasting blood glucose was elevated in HFD‐LiGHR^−/−^ mice (Figure S3F,G). GTT and ITT revealed impaired glucose tolerance and insulin sensitivity (Figure S3H,I), while PAS staining confirmed reduced hepatic glycogen synthesis capacity (Figure S3J). These results demonstrate that hepatic GHR deficiency exacerbates lipid deposition and systemic metabolic dysregulation under HFD conditions. To assess the impact of hepatic GHR deletion on HFD‐induced liver senescence, SA‐β‐Gal staining revealed increased β‐Gal activity in HFD‐LiGHR^−/−^ livers (Figure 2L). IHC and Western blot analyses confirmed upregulation of senescence markers (p16, p21, p53, and γH2AX) (Figure 2M,N). Fibrotic markers α‐SMA and Col1a1 were also elevated, as shown by Western blot (Figure S3K), and Masson's trichrome staining revealed increased collagen deposition (Figure S3L). Inflammatory markers IL6 and IL1β were further elevated in HFD‐LiGHR^−/−^ livers (Figure S3M). To further investigate the relationship between hepatic lipid accumulation and aging, we directly compared age‐related phenotypes in 12‐month‐old LiGHR^−/−^ mice under natural aging and HFD conditions (Figure S4A). HFD‐fed LiGHR^−/−^ mice exhibited markedly greater hepatic lipid accumulation than age‐matched chow‐fed LiGHR^−/−^ mice (Figure S4B). Moreover, the hepatic expression of senescence markers (p16, p21, and p53) and inflammatory factors (IL6 and TNFα) was significantly elevated in HFD‐fed LiGHR^−/−^ mice (Figure S4C,D). These findings indicate that HFD accelerates hepatic steatosis, inflammation, and cellular senescence in male LiGHR^−/−^ mice, further supporting a link between ectopic lipid accumulation and accelerated aging.

Additionally, AML12 cells were used to model hepatic senescence in vitro via lentiviral GHR knockdown. shRNA #3 exhibited maximal knockdown efficiency and was used in all subsequent experiments (Figure S5A). Cellular senescence was induced using FFA (Du et al. 2024; Gao et al. 2020), and GHR ablation synergized with FFA to enhance SA‐β‐Gal activity, indicating accelerated senescence (Figure 2O). Consistent with in vivo findings, FFA‐induced GHR‐deficient AML12 cells exhibited a more severe senescent phenotype, evidenced by the upregulation of p16, p21, and γH2AX (Figures 2P and S5B), along with decreased proliferation and migration (Figures 2Q and S5C). Oil Red O and BODIPY staining revealed increased cytosolic lipid droplet formation and TG accumulation in GHR‐deficient cells (Figure S5D,E). Furthermore, GHR knockdown potentiated SASP responses following FFA exposure, recapitulating the inflammatory phenotype observed in LiGHR^−/−^ mice (Figure S5F). Collectively, these data demonstrate that hepatocyte‐specific GHR deficiency exacerbates liver senescence, steatosis, and pro‐inflammatory activation both in vivo and in vitro.

### 3.3 Hepatocyte‐Specific GHR Deficiency Induces Adipose Lipolysis and Enhances Hepatic Lipid Uptake

**FIGURE 3:**
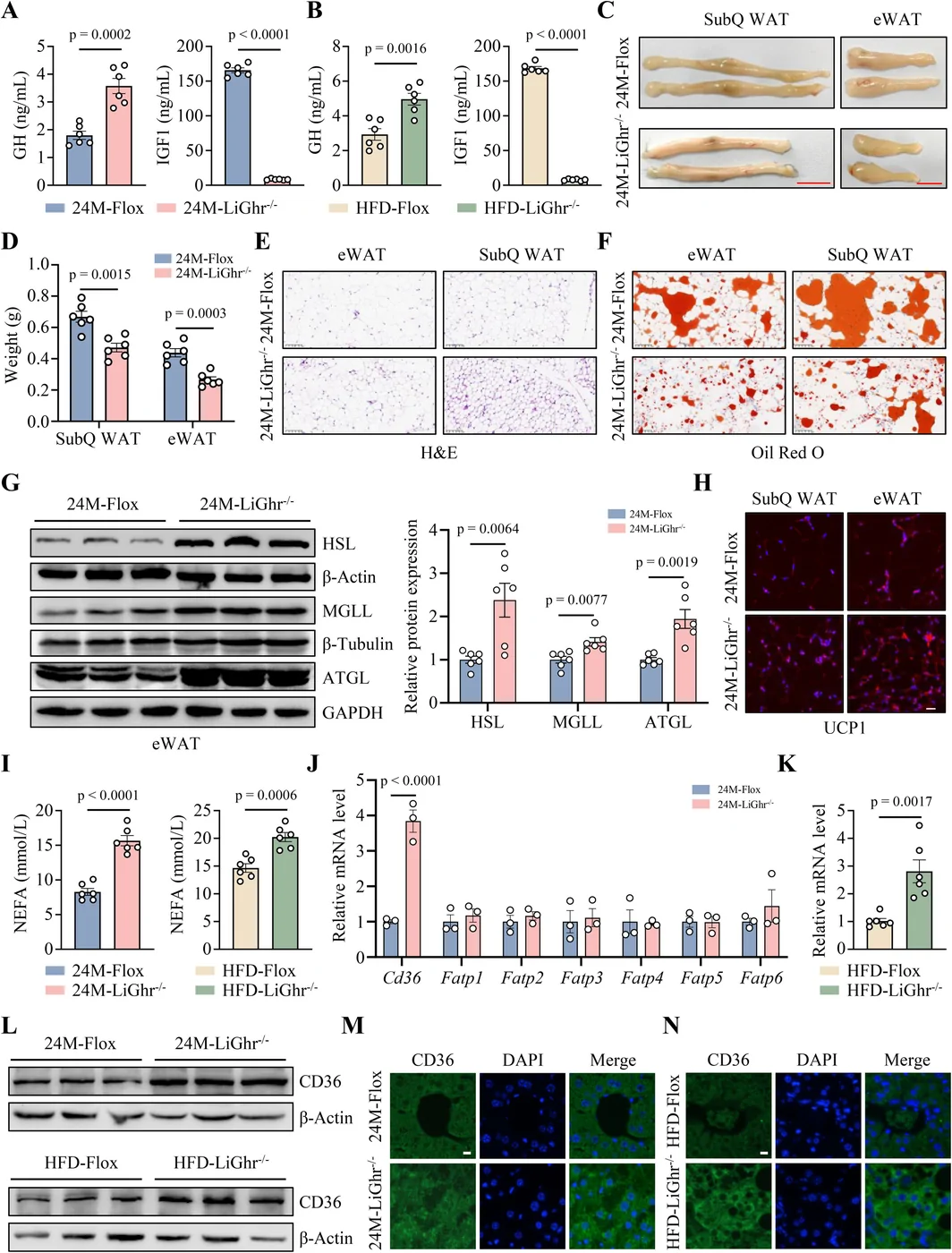
Hepatocyte‐specific GHR deficiency induces adipose lipolysis and enhances hepatic lipid uptake. (A) Serum GH and IGF1 levels in 24M‐Flox and 24M‐LiGHR^−/−^ male mice, *n* = 6. (B) Serum GH and IGF1 levels in HFD‐Flox and HFD‐LiGHR^−/−^ male mice, *n* = 6. (C) Images of SubQ WAT and eWAT from 24‐month‐old Flox and LiGHR^−/−^ mice. Scale bar: 1 cm. (D) Weights of SubQ WAT and eWAT from 24M‐Flox and 24M‐LiGHR^−/−^ mice, *n* = 6. (E) H&E staining of SubQ WAT and eWAT sections from 24M‐Flox and 24M‐LiGHR^−/−^ mice. Scale bar: 100 μm. (F) Oil Red O staining of SubQ WAT and eWAT sections from 24M‐Flox and 24M‐LiGHR^−/−^ mice. Scale bar: 100 μm. (G) Western blot analysis of lipolysis‐related proteins (HSL, ATGL, and MGLL) in eWAT from 24M‐Flox and 24M‐LiGHR^−/−^ mice, *n* = 6. (H) Immunofluorescence staining of UCP1 (red) in SubQ WAT and eWAT from 24M‐Flox and 24M‐LiGHR^−/−^ mice. Scale bar: 50 μm. (I) Serum NEFA levels in 24M‐Flox, 24M‐LiGHR^−/−^, HFD‐Flox and HFD‐LiGHR^−/−^ male mice, *n* = 6. (J) RT‐qPCR analysis of *Cd36* and *Fatp* family genes mRNA expression in the livers of 24M‐Flox and 24M‐LiGHR^−/−^ mice, *n* = 3. (K) The *Cd36* mRNA expression in HFD‐Flox and HFD‐LiGHR^−/−^ livers was detected, *n* = 6. (L) The CD36 protein level in livers of 24M‐Flox, 24M‐LiGHR^−/−^, HFD‐Flox, and HFD‐LiGHR^−/−^ mice was measured, *n* = 6. (M) Representative immunofluorescence images of CD36 (green) in livers of 24M‐Flox and 24M‐LiGHR^−/−^ mice. Scale bar: 25 μm. (N) Representative immunofluorescence images of CD36 (green) in livers of HFD‐Flox and HFD‐LiGHR^−/−^ mice. Scale bar: 25 μm.

To further investigate the role of hepatocyte‐specific GHR deficiency in aging, serum GH and IGF1 levels were measured in male Flox and LiGHR^−/−^ mice. GH levels were elevated and IGF1 levels were reduced in both 24M‐LiGHR^−/−^ and HFD‐LiGHR^−/−^ mice (Figure 3A,B). Given that GH promotes lipolysis in adipose tissue and regulates fat mass and distribution (Kopchick et al. 2020), we examined adipose depots. The weights of subcutaneous white adipose (SubQ WAT) and epididymal white adipose (eWAT) were reduced in 24M‐LiGHR^−/−^ mice compared to controls (Figure 3C,D). H&E, Oil Red O, and BODIPY staining confirmed decreased lipid droplet accumulation in SubQ WAT and eWAT of 24M‐LiGHR^−/−^ mice (Figures 3E,F and S6A). Similarly, long‐term HFD failed to increase SubQ WAT and eWAT mass in LiGHR^−/−^ mice (Figure S6B,C), and lipid content remained lower than in HFD‐Flox mice (Figure S6D,E). We next examined lipolytic and thermogenic protein expression. Lipolysis‐related proteins, including ATGL, HSL, and MGLL, were upregulated in eWAT of 24M‐LiGHR^−/−^ mice (Figure 3G). Concurrently, thermogenic marker UCP1 expression was increased (Figure 3H). Serum non‐esterified fatty acids (NEFA) were also elevated in LiGHR^−/−^ mice (Figure 3I). Collectively, hepatocyte‐specific GHR deficiency elevates circulating GH, enhancing adipose lipolysis, reducing fat mass, and increasing serum NEFA, which serves as a substrate for ectopic lipid deposition in the liver.

To determine how excess NEFA contributes to hepatic lipid accumulation, we measured hepatic mRNA levels of *Cd36* and *Fatp* family genes. Only *Cd36* mRNA expression was significantly upregulated in 24M‐LiGHR^−/−^ livers (Figure 3J), and similarly, HFD‐LiGHR^−/−^ livers exhibited increased *Cd36* mRNA expression (Figure 3K). Consistently, CD36 protein levels were elevated in both 24M‐LiGHR^−/−^ and HFD‐LiGHR^−/−^ livers (Figure 3L–N). These findings suggest that hepatocyte‐specific GHR deficiency promotes GH‐mediated lipolysis, increases circulating NEFA, and drives CD36‐dependent hepatic fatty acid uptake. The resulting lipid surplus contributes to ectopic lipid accumulation, inflammation, and accelerated hepatocyte senescence.

### 3.4 Hepatocyte‐Specific GHR Ablation Increases PPARγ Expression via p‐STAT5b Down‐Regulation

**FIGURE 4:**
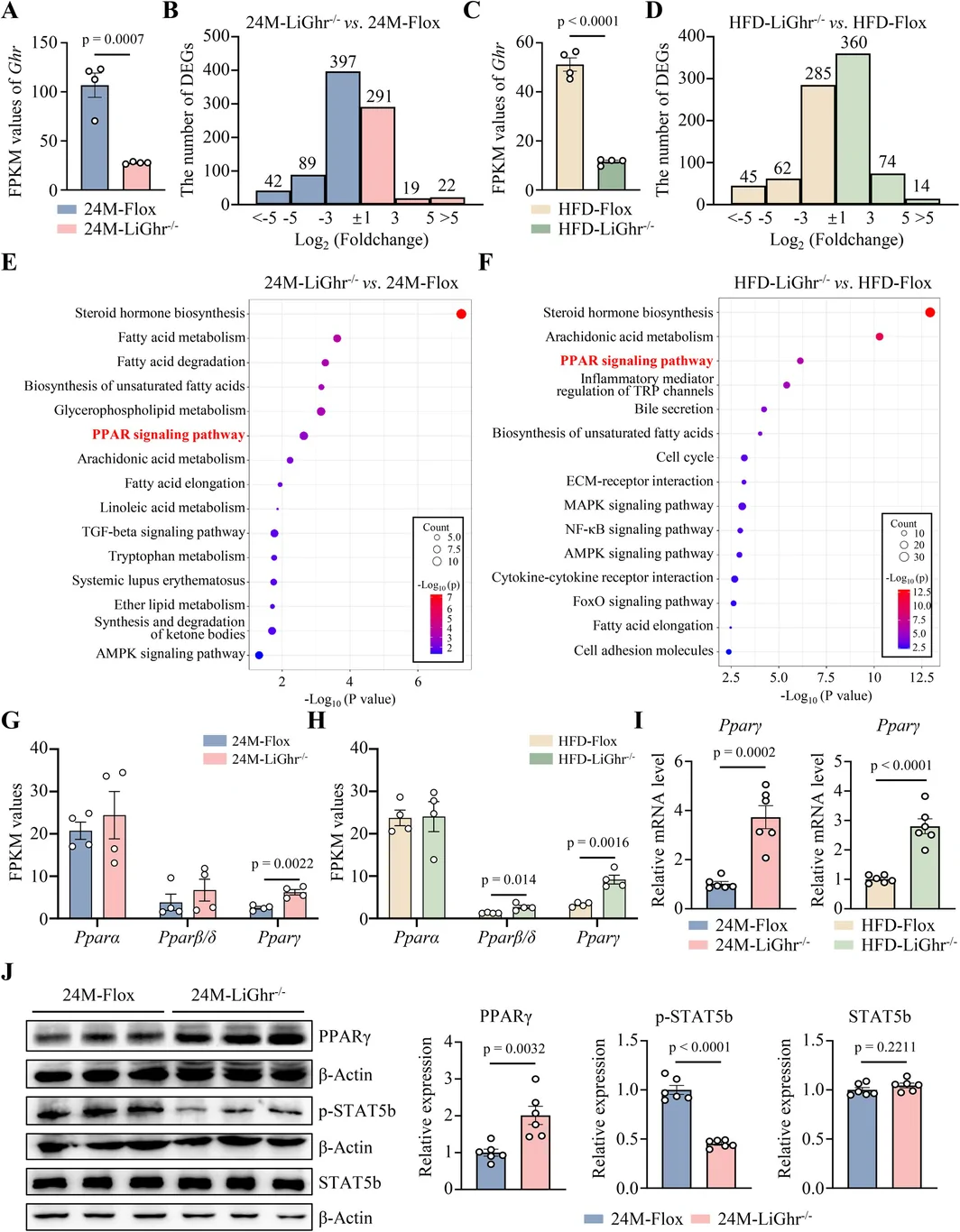
Hepatocyte‐specific GHR ablation exacerbates PPARγ expression via p‐STAT5b down‐regulation. (A) FPKM values of *Ghr* in livers of 24M‐Flox and 24M‐LiGHR^−/−^ mice, *n* = 4. (B) RNA‐seq analysis showing differentially expressed transcripts in livers of 24M‐LiGHR^−/−^ vs. 24M‐Flox mice. The number of transcripts in each fold‐change category is indicated above each bar. (C) FPKM values of *Ghr* in livers of HFD‐Flox and HFD‐LiGHR^−/−^ mice, *n* = 4. (D) RNA‐seq analysis showing differentially expressed transcripts in livers of HFD‐LiGHR^−/−^ vs. HFD‐Flox mice, with the number of transcripts in each fold‐change category indicated above each bar. (E) KEGG pathway enrichment analysis of DEGs in livers of 24M‐LiGHR^−/−^ mice compared to 24M‐Flox controls. (F) KEGG pathway enrichment analysis of DEGs in livers of HFD‐LiGHR^−/−^ mice compared to HFD‐Flox controls. (G) FPKM values of *Pparα*, *Pparβ/δ*, and *Pparγ* in liver of 24M‐Flox and 24M‐LiGHR^−/−^ mice. (H) FPKM values of *Pparα*, *Pparβ/δ*, and *Pparγ* in livers of HFD‐Flox and HFD‐LiGHR^−/−^ mice. (I) *Pparγ* mRNA expression in mouse livers from different groups was measured, *n* = 6. (J) PPARγ, STAT5b, and p‐STAT5b protein levels in livers of 24M‐Flox and 24M‐LiGHR^−/−^ mice were measured, *n* = 6.

To investigate the molecular consequences of hepatocyte‐specific GHR ablation, RNA sequencing was performed on liver tissues from male 24‐month‐old and HFD‐fed LiGHR^−/−^ mice and their respective Flox controls. As expected, hepatic *Ghr* and *Igf1* mRNA expression was decreased in both aged LiGHR^−/−^ mice and HFD‐fed LiGHR^−/−^ mice (Figures 4A,C and S7A,C). RT‐qPCR further confirmed that *Igf1* mRNA levels were reduced in LiGHR^−/−^ mice (Figure S7B,D). Principal component analysis (PCA) revealed clear clustering of samples by genotype and treatment (Figure S7E,G). Differential expression analysis identified 860 significantly altered transcripts in 24M‐LiGHR^−/−^ versus 24M‐Flox livers (528 down‐regulated and 332 up‐regulated; Figures 4B and S7F), and 840 DEGs in HFD‐LiGHR^−/−^ versus HFD‐Flox livers (392 down‐regulated, 448 up‐regulated; Figures 4D and S7H). Gene enrichment analysis indicated that the DEGs were predominantly involved in fatty acid metabolism, inflammatory signaling, PPAR signaling pathway, AMPK signaling pathway and NF‐κB signaling pathway (Figure 4E,F), highlighting the role of hepatocyte‐specific GHR deficiency in promoting hepatic steatosis and inflammatory activation. The mTOR, SIRT1/5, NF‐κB, and AMPK signaling pathways are key regulators of aging and metabolism and may contribute to the accelerated aging phenotype observed in LiGHR^−/−^ mice. We therefore examined these pathways in the livers of aged male LiGHR^−/−^ and Flox mice. Although SIRT5 expression was unchanged, p‐AMPK and SIRT1 levels were significantly reduced, whereas p‐mTOR and p‐P65 levels were markedly increased in LiGHR^−/−^ mice (Figure S7I–L). These findings suggest that multiple aging‐related signaling pathways are dysregulated and may collectively contribute to accelerated aging in male LiGHR^−/−^ mice.

Notably, dysregulation of the PPAR signaling pathway is a key factor in liver pathology (Li et al. 2025). In this study, RNA‐seq data revealed marked upregulation of PPARγ in both 24M‐ and HFD‐LiGHR^−/−^ livers, whereas PPARα and PPARβ/δ remained unchanged (Figure 4G,H). The elevated PPARγ expression observed in both 24M‐ and HFD‐LiGHR^−/−^ livers was further supported by RT‐qPCR and Western blot (Figures 4I,J and S7M). GH signaling normally activates p‐STAT5b, which translocates to the nucleus to regulate target gene transcription. Disruption of GH‐GHR signaling leads to p‐STAT5b inactivation and dysregulation of downstream genes (Kaltenecker et al. 2019). Consistently, p‐STAT5b expression was significantly reduced in 24M‐ and HFD‐LiGHR^−/−^ livers (Figures 4J and S7M). Reduced p‐STAT5b relieves transcriptional suppression of PPARγ, consistent with in vitro evidence of reciprocal regulation between p‐STAT5b and PPARγ (Shipley and Waxman 2003; Wakao et al. 2011). Collectively, these results demonstrate that hepatocyte‐specific GHR ablation attenuates p‐STAT5b signaling, thereby enhancing PPARγ expression and contributing to metabolic dysregulation.

### 3.5 Elevated PPARγ Promotes PDK4 Expression in Mice With Hepatocyte‐Specific GHR Deficiency

**FIGURE 5:**
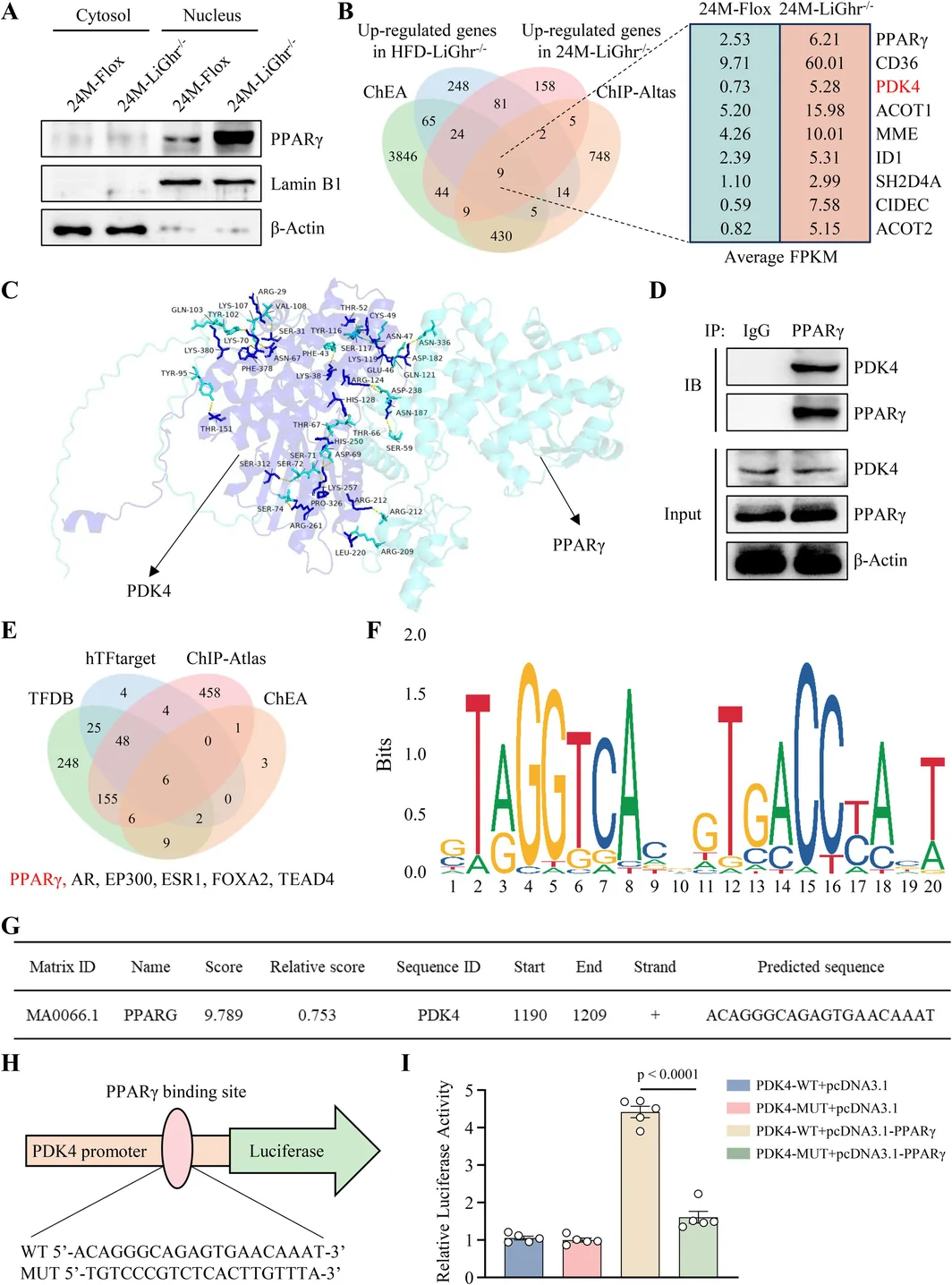
Elevated PPARγ promotes PDK4 expression in mice with hepatocyte‐specific GHR deficiency. (A) Liver tissues from 24M‐Flox and 24M‐LiGHR^−/−^ mice were fractionated into nuclear and non‐nuclear fractions. PPARγ, β‐Actin, and Lamin B1 protein levels were analyzed by Western blot. (B) Venn diagram showing the overlap of upregulated genes in the liver of HFD‐fed LiGHR^−/−^ mice, upregulated genes in 24‐month‐old LiGHR^−/−^ mice, and PPARγ target genes from the ChEA and ChIP‐Atlas database. Nine overlapping genes were identified as potential direct targets of PPARγ in aged LiGHR^−/−^ liver. The right panel displays the average FPKM values of these nine genes (PPARγ, CD36, PDK4, ACOT1, MME, ID1, SH2D4A, CIDEC, and ACOT2) in 24M‐Flox and 24M‐LiGHR^−/−^ mice. (C) Molecular docking between PPARγ and PDK4. Protein sequences were obtained from UniProt (https://www.uniprot.org/), and potential binding sites were predicted using AlphaFold3 and visualized with PyMOL. (D) Co‐immunoprecipitation (Co‐IP) of PPARγ in liver tissues from 24M‐LiGHR^−/−^ mice. Immunoblots (IB) were probed with anti‐PDK4, anti‐PPARγ, and anti‐β‐Actin. *n* = 3. (E) Venn diagram showing six potential transcription factors for PDK4 identified from TFDB, hTFtarget, ChIP‐Atlas, and ChEA databases. (F) Schematic of the PPARγ binding motif (MA0066.1, JASPAR) and predicted PPARγ‐responsive sequence (ACAGGGCAGAGTGAACAAAT) within the PDK4 promoter. (G) Predicted binding of PPARγ to the PDK4 promoter using the JASPAR database. (H) Schematic model of wild type (WT) and mutant (MUT) sequences of two putative binding sites of PPARγ on PDK4 promoter. (I) The relative luciferase activities were detected in HEK293T cells co‐transfected with luciferase reporter plasmids containing wild type or mutant PDK4 promoter sequence and pcDNA3.1 or pcDNA3.1‐PPARγ (*n* = 5).

PPARγ is a well‐established transcriptional regulator that drives hepatic pathogenesis; however, the mechanistic link between hepatocyte‐specific GHR ablation‐induced PPARγ activation and accelerated aging remains unclear. Subcellular fractionation revealed enhanced nuclear translocation of PPARγ in 24M‐LiGHR^−/−^ liver compared to 24M‐Flox controls, indicating increased transcriptional activity (Figure 5A). To identify downstream targets, PPARγ target genes from ChEA and ChIP‐Atlas databases were cross‐referenced with hepatic transcriptomic profiles from 24M‐ and HFD‐LiGHR^−/−^ mice. Venn analysis highlighted CD36, PDK4, ACOT1, MME, ID1, SH2D4A, CIDEC, and ACOT2 as upregulated PPARγ targets, while RARRES1, HSD17B2, and CAPN8 were downregulated (Figures 5B and S8A), with *Pdk4* mRNA exhibiting a notable increase of over sevenfold in 24M‐LiGHR^−/−^ livers. Further analysis using AlphaFold3 predicted multiple interaction sites between PPARγ and PDK4 (Figure 5C). Co‐immunoprecipitation confirmed PPARγ physically interacts with PDK4 in 24M‐LiGHR^−/−^ livers (Figure 5D). These results suggest that PPARγ mediates PDK4 activation.

To elucidate the mechanism of PPARγ‐dependent PDK4 transcription, bioinformatic analysis identified potential transcription factors binding the PDK4 promoter, including PPARγ, AR, EP300, ESR1, FOXA2, and TEAD4 (Figure 5E). RNA sequencing revealed no significant changes in AR, EP300, ESR1, FOXA2, or TEAD4 expression in 24M‐ or HFD‐LiGHR^−/−^ livers (Figure S8B,C). JASPAR database predictions indicated potential PPARγ binding sites within the PDK4 promoter (Figure 5F,G). Dual‐luciferase reporter assays using wild‐type and mutant PDK4 promoters confirmed PPARγ‐dependent transcriptional activation. Mutation of the predicted PPARγ binding motif abolished luciferase activity, confirming specific promoter binding (Figure 5H,I).

To further investigate whether reduced p‐STAT5b relieves inhibition of PPARγ and subsequently activates PDK4, we generated STAT5b‐overexpressing (STAT5b‐OE) cells and confirmed markedly increased STAT5b protein expression (Figure S8D). STAT5b expression significantly reduced the protein levels of both PPARγ and PDK4, supporting a regulatory axis in which hepatocyte‐specific GHR deletion decreases p‐STAT5b, leading to upregulation of PPARγ and PDK4. To further validate this pathway, PPARγ was silenced in AML12 cells. Among the three siRNAs tested, siRNA #3 showed the highest knockdown efficiency and was used for subsequent experiments (Figure S8E). PPARγ knockdown significantly reduced both PDK4 mRNA and protein expression levels (Figure S8F,G), confirming that PPARγ positively regulates PDK4 expression. To determine whether GH directly regulates CD36 and PDK4 expression in hepatocytes, control and GHR‐knockdown (shGHR) AML12 cells were treated with GH. Consistent with the in vivo findings, GHR knockdown increased CD36 and PDK4 expression following FFA treatment. In control cells, GH treatment suppressed CD36 expression while enhancing PDK4 expression (Figure S8H), suggesting that GH regulates these genes through distinct downstream mechanisms when GHR signaling is intact. Importantly, these effects were abolished in shGHR cells, demonstrating that GH‐mediated regulation of CD36 and PDK4 is dependent on GHR signaling. Collectively, these results further support the involvement of the GH‐GHR‐STAT5b‐PPARγ‐PDK4 axis in hepatic lipid metabolism and aging.

### 3.6 Hepatocyte‐Specific GHR Ablation Drives Mitochondrial Dysfunction via PDK4 Upregulation

**FIGURE 6:**
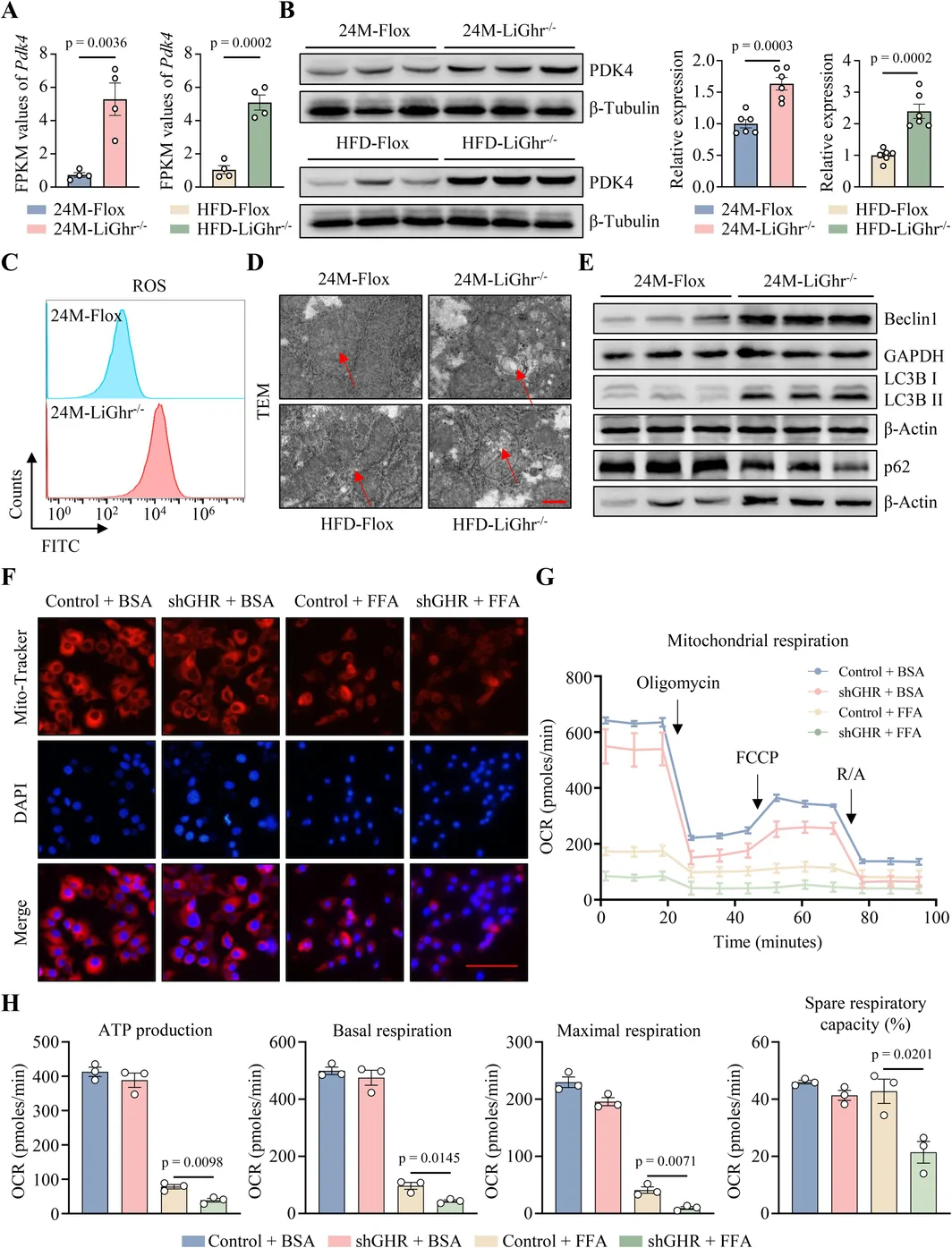
Hepatocyte‐specific GHR ablation drives mitochondrial dysfunction via PDK4 upregulation. (A) FPKM values of PDK4 in liver tissues from 24M‐Flox, 24M‐LiGHR^−/−^, HFD‐Flox and HFD‐LiGHR^−/−^ mice. *n* = 4. (B) The PDK4 protein expression in the livers of 24M‐Flox, 24M‐LiGHR^−/−^, HFD‐Flox, and HFD‐LiGHR^−/−^ mice was examined. *n* = 6. (C) ROS levels in liver tissues from 24M‐Flox and 24M‐LiGHR^−/−^ mice were measured by flow cytometry. (D) Representative TEM images of liver tissues from 24M‐Flox, 24M‐LiGHR^−/−^, HFD‐Flox, and HFD‐LiGHR^−/−^ mice. Mitochondrial defects are indicated by red arrows. Scale bar: 2 μm. (E) Western blot analysis of Beclin1, LC3B‐II/LC3B‐I ratio, and p62 protein levels in liver tissues from 24M‐Flox and 24M‐LiGHR^−/−^ mice. *n* = 6. (F) Representative Mito‐Tracker staining in control and shGHR cells treated with BSA or FFA. Scale bar: 50 μm. (G) Mitochondrial OCR in control and shGHR cells treated with BSA or FFA was measured using the Seahorse XF Analyzer. *n* = 3. (H) Quantification of basal respiration, maximal respiratory capacity, ATP production, and spare respiratory capacity in control and shGHR cells treated with BSA or FFA, measured using the Seahorse XF Analyzer.

RNA sequencing revealed that *Pdk4* mRNA levels were increased in the livers of 24M‐ and HFD‐LiGHR^−/−^ mice (Figure 6A). Western blot confirmed robust upregulation of PDK4 protein in these livers (Figure 6B). Consistent with prior reports, PDK4 is elevated in multiple hepatic metabolic disorders (Hwang et al. 2009; Thoudam et al. 2023; Zhang, Zhao, et al. 2018), and its inhibition has been shown to reduce fatty acid‐induced mitochondrial Ca^2+^ overload and reactive oxygen species (ROS) production, thereby ameliorating mitochondrial dysfunction (Thoudam et al. 2019). To determine whether hepatic GHR deficiency induces mitochondrial dysfunction, we first measured ROS levels, which were elevated in livers of 24M‐ and HFD‐LiGHR^−/−^ mice (Figures 6C and S9A). TEM revealed severe mitochondrial ultrastructural abnormalities, including disrupted membranes and disorganized cristae (Figure 6D). The antioxidant defense system was impaired in the liver of aged LiGHR^−/−^ mice, as demonstrated by significantly reduced activities of superoxide dismutase (SOD), catalase (CAT), and glutathione peroxidase (GSH‐Px) (Figure S9B). These findings indicate that hepatocyte‐specific GHR deletion disrupts redox homeostasis and promotes oxidative stress during aging. Moreover, core autophagy markers, such as Beclin1 and the LC3B‐II/LC3B‐I ratio, were upregulated, while p62/SQSTM1 was downregulated, indicating activated autophagic flux (Figures 6E and S9C). To assess the functional consequences of GHR deficiency, mitochondrial bioenergetics were analyzed in FFA‐challenged GHR knockdown AML12 cells. Mito‐Tracker Red staining revealed pronounced mitochondrial morphological defects, and OCR measurements demonstrated that GHR ablation synergistically exacerbated FFA‐induced suppression of mitochondrial respiratory capacity (Figure 6F–H). Collectively, these data indicate that hepatocyte‐specific GHR ablation drives PDK4 upregulation, leading to mitochondrial dysfunction, which in turn contributes to hepatic senescence, pro‐inflammatory signaling activation, and ectopic lipid accumulation.

### 3.7 Targeting PDK4 Alleviates Age‐Related Pathologies Driven by Hepatocyte‐Specific GHR Ablation

**FIGURE 7:**
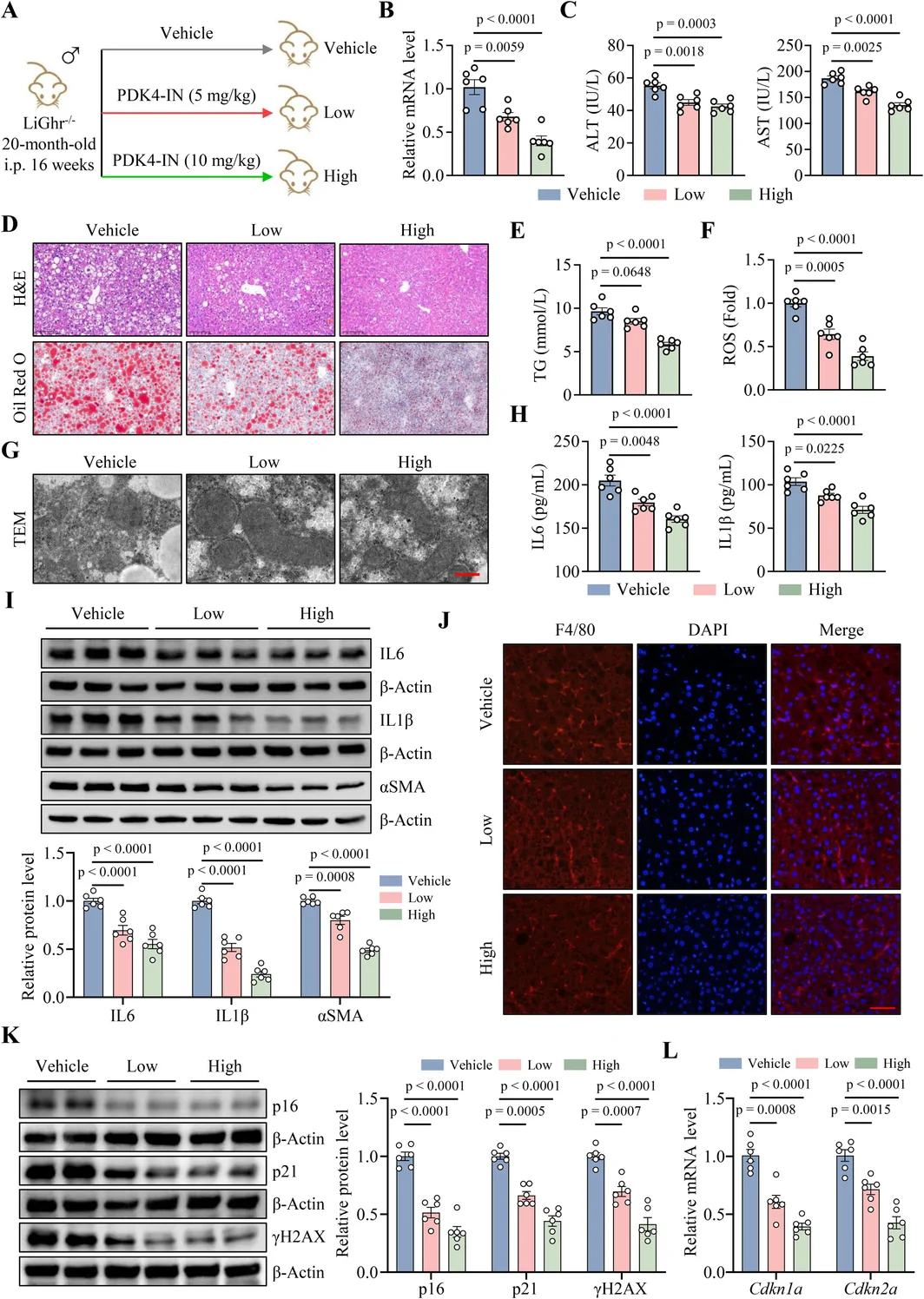
Targeting PDK4 ameliorates age‐related pathologies induced by hepatocyte‐specific GHR ablation. (A) Schematic of the experimental design. 20‐month‐old male LiGHR^−/−^ mice were treated with vehicle or PDK4‐IN (5 or 10 mg/kg, i.p., weekly) for 16 consecutive weeks (*n* = 6 per group). (B) *Pdk4* mRNA levels in the livers of LiGHR^−/−^ mice treated with vehicle or PDK4‐IN (5 or 10 mg/kg) were measured. (C) Serum levels of ALT and AST in LiGHR^−/−^ mice treated with vehicle or PDK4‐IN (5 or 10 mg/kg) were measured by ELISA. (D) Representative images of H&E (Scale bar: 100 μm) and Oil Red O (Scale bar: 50 μm) staining of liver sections from LiGHR^−/−^ mice treated with vehicle or PDK4‐IN (5 or 10 mg/kg). (E) Serum TG levels in mice from different groups were measured by ELISA. (F) ROS levels in liver tissues from LiGHR^−/−^ mice treated with vehicle or PDK4‐IN (5 or 10 mg/kg) were measured. (G) Representative TEM images of liver tissues from vehicle‐ or PDK4‐IN‐treated LiGHR^−/−^ mice. Scale bar: 2 μm. (H) Hepatic IL6 and IL1β levels in LiGHR^−/−^ mice from different groups were measured by ELISA. (I) Protein levels of IL6, IL1β, and αSMA in the livers of LiGHR^−/−^ mice treated with vehicle or PDK4‐IN (5 or 10 mg/kg) were measured. (J) Immunofluorescence staining of F4/80 in liver tissues from different groups. Macrophages are labeled with F4/80 (red) and nuclei with DAPI (blue). Scale bar: 50 μm. (K) Protein levels of p16, p21, and γH2AX in the livers of LiGHR^−/−^ mice treated with vehicle or PDK4‐IN (5 or 10 mg/kg) were measured. (L) The mRNA levels of *Cdkn1a* and *Cdkn2a* in livers of LiGHR^−/−^ mice treated with vehicle or PDK4‐IN (5 or 10 mg/kg) were detected.

PDK4 serves as a key factor in promoting the vicious circle of lipid ectopic deposition and mitochondrial dysfunction, which in turn accelerates aging in hepatocyte‐specific GHR‐ablated mice. Subsequently, we administered PDK4‐IN to investigate whether it could delay or inhibit the age‐related phenotypes caused by hepatocyte‐specific GHR ablation. In this study, we treated 20‐month‐old LiGHR^−/−^ and Flox mice with either the vehicle or PDK4‐IN at 5 mg/kg (Low) or 10 mg/kg (High) once weekly for 16 weeks (Figures 7A and S10A). The levels of PDK4 were markedly reduced in the livers of LiGHR^−/−^ mice from both the Low and High dose groups (Figure 7B). Compared with the vehicle group, serum ALT and AST levels were decreased in PDK4‐IN groups, indicating that PDK4 inhibition could ameliorate liver injury (Figures 7C and S10B). Aged LiGHR^−/−^ mice exhibit liver dysfunction and marked lipid deposition. Notably, administration of PDK4‐IN significantly prevented these alterations, indicating that PDK4 inhibition alleviates liver pathology and demonstrates the feasibility of targeting senescent cells to control this pathology (Figure 7D,E). Mitochondrial ultrastructural abnormalities, including disrupted membranes and disorganized cristae, were alleviated in the liver of LiGHR^−/−^ mice from both the Low and High groups, which were basically in line with reduced ROS levels in the liver (Figure 7F,G). In addition, we measured inflammatory factors in the blood and liver tissue, as well as hepatic fibrosis, following PDK4‐IN treatment. The levels of IL6 and IL1β in the serum decreased significantly in a dose‐dependent manner (Figure 7H). Moreover, hepatic fibrosis and inflammation were markedly alleviated (Figure 7I,J). Together, PDK4‐IN treatment partially ameliorates the liver pathology induced by hepatocyte‐specific GHR ablation. More importantly, expression of age‐related markers (p16, p21, and γH2AX) was downregulated in liver tissues from the PDK4‐IN treatment groups, indicating that inhibition of PDK4 relieves hepatocyte growth arrest and DNA damage accumulation (Figure 7K,L). Furthermore, we evaluated whether PDK4‐IN exerts similar anti‐aging effects in naturally aged Flox mice. PDK4‐IN treatment significantly reduced the hepatic expression of senescence‐ and inflammation‐related proteins (Figure S10C,D), indicating that PDK4 inhibition can alleviate age‐associated liver dysfunction and may exert broader anti‐aging effects. Together, these results show that targeting PDK4 attenuates hepatocyte‐specific GHR ablation‐induced age‐related pathological damage, demonstrating that this therapeutic strategy alleviates hepatic pathological damage and delays markers of age‐related liver degeneration, supporting further investigation of PDK4 as a potential target for extending healthspan.

## 4 Discussion

GH binding to GHR activates downstream signaling pathways that coordinate somatic development and metabolic homeostasis, serving as key regulators of mammalian lifespan (Guevara‐Aguirre et al. 2018; Poudel et al. 2025). Our recent findings indicate that targeted deletion of GHR in adipocytes induces protective adipose remodeling, enhances systemic metabolic tolerance, and remotely alleviates brain aging and cognitive decline, without the developmental complications associated with global GHR deficiency (Ma et al. 2026; Zhang et al. 2026). Together, these findings suggest adipose GHR as a tissue‐specific regulator of healthy aging and a promising therapeutic target for age‐related metabolic and cognitive decline. However, the relationship between hepatic GHR and age‐related pathologies remains poorly understood. In this study, hepatocyte‐specific GHR deficiency is associated with accelerated aging‐related phenotypes, including cognitive decline, pro‐inflammatory activation, and age‐related liver degeneration. Mechanistically, hepatocyte‐specific GHR ablation elevates circulating GH, thereby promoting adipose lipolysis and ectopic lipid accumulation, which ultimately leads to systemic metabolic dysregulation. Furthermore, hepatocyte‐specific GHR ablation attenuates STAT5b phosphorylation, leading to upregulation of PPARγ. Enhanced nuclear translocation of PPARγ transcriptionally activates PDK4, inducing mitochondrial dysfunction and ROS generation. Collectively, lipid metabolism dysregulation and mitochondrial impairment engage in a reciprocal pathological loop, synergistically driving aging phenotypes. Treatment with the PDK4 inhibitor (PDK4‐IN) attenuates hepatocyte‐specific GHR ablation‐induced age‐related pathological damage.

The liver orchestrates systemic metabolic homeostasis through energy regulation, xenobiotic detoxification, and synthesis of essential molecules. Age‐associated hepatic alterations increase susceptibility to geriatric pathologies, including insulin resistance, hepatic steatosis, inflammation, and impaired organ function (Yang et al. 2023). Senescent hepatocytes drive extrahepatic organ degeneration (affecting kidneys, brain, and lungs) and induce steatosis, ultimately leading to marked hepatic accumulation of TG and T‐CHO (Duan et al. 2023; Kiourtis et al. 2024). Moreover, decreased PPARα in the liver tissue of aged mice results in impaired mitochondrial β‐oxidation, which contributes to hepatic lipid accumulation in these mice (Wan et al. 2020). In patients with metabolic dysfunction‐associated fatty liver disease (MAFLD), increased hepatic lipid accumulation correlates with upregulation of p16 expression in hepatocytes (Zhang, Xu, et al. 2018). Consistent with this observation, sustained HFD feeding in experimental models further demonstrates that lipid overload significantly induces both p16 expression and the release of SASP components, implying that lipid‐driven hepatocyte senescence may contribute to disease progression (Kennedy et al. 2021; Zhang, Xu, et al. 2018). Senescent hepatocytes and ectopic lipid deposition form a mutually reinforcing pathological cycle, in which the accumulation of senescent cells exacerbates hepatic lipid deposition, whereas clearance of these cells using dasatinib and quercetin alleviates steatosis (Du et al. 2024; Ogrodnik et al. 2017). In LiGHR^−/−^ male mice, both natural aging and HFD‐induced stress resulted in accelerated hepatic aging, characterized by lipid deposition, inflammatory activation, and fibrogenesis, partially mediated by CD36‐facilitated NEFA trafficking. The aging trajectories of LiGHR^−/−^ female mice were not significantly affected, likely due to hormonal fluctuations.

GH stimulation creates a STAT5b docking site in the GHR‐JAK2 complex, triggering its tyrosine phosphorylation in the cytoplasm and ultimately the activation of specific target gene transcription, which is critical for liver‐specific gene expression, growth, and metabolic regulation (Hwa 2021). Hepatocyte‐specific GHR knockout reduces STAT5b phosphorylation, impairing its transcriptional activity. STAT5b inhibits PPARγ‐mediated transcription, whereas PPARγ reciprocally suppresses STAT5b‐dependent transcriptional activity in a dose‐dependent manner (Shipley and Waxman 2003). PPARγ plays a central role in regulating aging, glucose homeostasis, inflammation, and lipogenesis (Li et al. 2025). Specifically, PPARγ activation upregulates key senescence markers (p16 and p27) and stimulates the synthesis of oxygenated lipids in bone marrow adipocytes. Furthermore, studies indicate that PPARγ promotes cellular senescence in human diploid fibroblasts by inducing p16 expression, accompanied by elevated SA‐β‐gal activity and reduced cell proliferation (Gan et al. 2008; Liu et al. 2023). In this study, the reciprocal inhibition between STAT5b and PPARγ is disrupted upon hepatocyte‐specific GHR ablation, where reduced STAT5b phosphorylation relieves transcriptional suppression of PPARγ. Enhanced PPARγ activity promotes CD36 expression and perturbs lipid homeostasis. PPARγ also transcriptionally activates PDK4, a key regulator of mitochondrial metabolism (Jeon et al. 2021; Thoudam et al. 2019). Elevated PDK4 disrupts mitochondrial function by inhibiting the pyruvate dehydrogenase complex, promoting ROS production, and impairing energy homeostasis (Ma et al. 2020; Thoudam et al. 2023). These processes collectively accelerate hepatocyte senescence, systemic inflammation, and ectopic lipid deposition.

Hepatic PDK4 levels are robustly induced in patients with MAFLD and in oleic acid‐treated hepatocytes. Consistent with this upregulation, deficiency of *Pdk4* significantly ameliorates hepatic steatosis in NASH mice (Hwang et al. 2009; Zhang, Zhao, et al. 2018). Obesity‐induced elevation of PDK4 activity promotes the formation of mitochondria‐associated endoplasmic reticulum membranes, leading to impaired insulin signaling. Inhibition of PDK4 prevents mitochondrial dysfunction and improves insulin response (Thoudam et al. 2019). In atherosclerosis, PDK4 upregulation drives vascular calcification by inducing mitochondrial dysfunction, thereby reducing mitochondrial respiratory capacity (Ma et al. 2020). Mitochondrial dysfunction is a hallmark of aging, contributing to reduced ATP production, oxidative stress, accumulation of ROS, and metabolic reprogramming (Dabravolski et al. 2021). In the present study, PDK4‐mediated impairment of mitochondrial bioenergetics further amplifies ROS generation, lipid accumulation, and senescence signaling. Sustained NEFA excess compounds mitochondrial stress, creating a pathogenic feedback loop between lipid dysregulation and mitochondrial dysfunction. According to a previous study (Dou et al. 2023), PDK4‐IN treatment significantly reduced serum ALT, AST, and lactate dehydrogenase levels in aged mice without affecting body weight or food intake, indicating a favorable safety profile. Histological analyses further showed reduced SA‐β‐gal‐positive cells in multiple organs, including the liver, lung, prostate, and heart, along with improvements in physical performance, such as grip strength, motor coordination, balance endurance, and daily activity. Notably, late‐life administration of PDK4‐IN increased median lifespan by 29.6% and maximum lifespan by 4.2%, while reducing mortality. Although the effects of PDK4‐IN on adipose tissue and skeletal muscle tissue were not evaluated, its broad systemic benefits suggest potential protective effects across multiple metabolic organs. Consistent with these findings, PDK4‐IN treatment in our study significantly reduced liver injury, lipid accumulation, inflammation, and senescence marker expression, while restoring mitochondrial structure in aged LiGHR^−/−^ mice. Together, these results indicate that targeting PDK4 effectively attenuates age‐related pathology and may represent a promising therapeutic strategy for liver aging, with possible broader relevance pending further study in extrahepatic tissues.

We acknowledge an important limitation inherent to the LiGHR^−/−^ model. Because the Cre‐mediated GHR deletion in this model is restricted to hepatocytes, all other tissues retain intact GHR expression and remain fully responsive to the elevated circulating GH due to the hepatocyte‐specific GHR loss and the consequent disruption of the GH‐GHR feedback axis. Therefore, this model cannot fully distinguish the direct, hepatocyte‐specific consequences caused by the hepatocyte‐specific GHR loss from the indirect effects caused by chronically elevated GH acting on the intact peripheral tissues (including adipose tissue, skeletal muscle, bone, and the brain). The extent to which each of these compartments contributes to systemic phenotypes such as cognitive decline, reduced bone mineral density, and shortened lifespan cannot be determined from the current experimental design. Resolving this question will require models that circumvent the confound intrinsic to constitutive hepatocyte‐specific GHR deletion, such as tamoxifen‐inducible hepatocyte‐specific GHR deletion in adult mice to avoid developmental compensation, or a reversible knockout strategy, such as the doxycycline/Tet‐Off system (Eguchi et al. 2026), in which hepatic GHR expression can be switched off and on to define the specific contribution of hepatocyte‐specific GHR loss to systemic aging.

## 5 Conclusion

The liver plays a central role in regulating lipid metabolism, mitochondrial function, and oxidative stress to maintain systemic homeostasis. Aging disrupts these functions, promoting a pathogenic secretome that accelerates senescence and organ dysfunction. Our findings demonstrate that hepatocyte‐specific GHR deficiency perturbs hepatic lipid metabolism and mitochondrial homeostasis, drives liver‐intrinsic senescence, and is associated with broader phenotypes including elevated inflammation, impaired cognitive function, and shortened lifespan in aged mice. Because this model does not allow the liver‐intrinsic consequences of GHR loss to be fully separated from the effects of elevated circulating GH on GHR‐intact peripheral tissues, we interpret these systemic findings as indicating that hepatocyte‐specific GHR ablation contributes to, rather than singularly drives, the observed aging phenotype. Within the liver itself, our data support the STAT5b‐PPARγ‐PDK4 axis as a mechanistic driver of hepatic senescence and steatosis, and identify hepatic PDK4 as a potential therapeutic target for mitigating age‐related liver disease.

## Author Contributions

Yingjie Wu and Shujing Wang contributed to the conception of research and study design and critically revised the article for important intellectual content. Kangkang Yang conducted the experimental work, wrote the original manuscript, and analyzed data. Yuli Jian, Fusheng Pang, Ming Ying, Qian Yang, and Nian Liu assisted in the experiment. All authors reviewed the results and approved the final version of the manuscript.

## Funding

This research was funded by the National Key Research and Development Program of China (No. 2021YFA0805100) and the National Natural Science Foundation of China (No. 82370866).

## Conflicts of Interest

The authors declare no conflicts of interest.

## Supporting information


**Figure S1:** Generation and characterization of hepatocyte‐specific GHR knockout mice. (A) Schematic illustration of LiGHR^−/−^ mouse construction. (B) PCR genotyping of Flox and LiGHR^−/−^ mice. (C) PCR analyses of GHR expression in various tissues (liver, subcutaneous white adipose tissue (SubQ), epididymal white adipose tissue (Epi), perirenal white adipose tissue (Peri), mesenteric white adipose tissue (Mes), brown adipose tissue (BAT), heart, spleen, kidney, lung, brain, and small intestine). A truncated band resulting from Cre‐mediated excision was detected exclusively in LiGHR^−/−^ liver tissue. (D) Survival curves for female Flox (*n* = 22) and LiGHR^−/−^ (*n* = 20) mice. (E) Glucose tolerance tests (GTT) in 24M male Flox and LiGHR^−/−^ mice, *n* = 6. (F) Insulin tolerance tests (ITT) in the indicated groups, *n* = 6.
**Figure S2:** Hepatocyte‐specific GHR ablation exacerbates hepatic histopathology in male LiGHR^−/−^ mice. (A) Representative SA‐β‐gal staining images of major metabolic tissues (brain, subcutaneous white adipose tissue (SubQ WAT), epididymal white adipose tissue (eWAT), kidney, and heart) from 24M‐Flox and 24M‐LiGHR^−/−^ mice. (B) Images of livers from 24M male Flox and LiGHR^−/−^ mice. Scale bar: 1 cm. (C) Liver weights of 24‐month‐old male Flox and LiGHR^−/−^ mice, *n* = 9. (D) Liver‐to‐body weight ratios of the indicated groups (*n* = 9 per group). (E) Immunohistochemical detection of γH2AX in the liver of 24M‐Flox and 24M‐LiGHR^−/−^ mice. Scale bar: 50 μm. (F) Immunofluorescence staining for α‐SMA in liver tissues from 24M‐Flox and 24M‐LiGHR^−/−^ mice. Scale bar: 50 μm. (G) Western blot analysis of IL1β and IL6 protein levels in liver tissues of 24M‐Flox and 24M‐LiGHR^−/−^ mice. (H) Schematic illustration of the experimental design. Male LiGHR^−/−^ mice were fed regular chow and aged to 6, 12, or 24 months before sacrifice for subsequent analyses (*n* = 4 per group). (I) Representative Oil Red O staining images of liver sections from male LiGHR^−/−^ mice at 6, 12, or 24 months of age. Scale bar: 100 μm. (J) Western blot analysis of senescence‐associated proteins p16 and p53 in liver tissues from male LiGHR^−/−^ mice at different ages. (K) Western blot analysis of inflammatory markers TNFα and IL6 in liver tissues from male LiGHR^−/−^ mice at different ages.
**Figure S3:** Hepatocyte‐specific GHR deficiency potentiates HFD‐induced blood glucose disorder and hepatic injury. (A) Body weights of male HFD‐Flox and HFD‐LiGHR^−/−^ mice (*n* = 6 per group). (B) Representative images of H&E (Scale bar: 200 μm) and Oil Red O (Scale bar: 100 μm) staining of liver sections from HFD‐Flox and HFD‐LiGHR^−/−^ mice. (C) Liver‐to‐body weight ratios of HFD‐Flox and HFD‐LiGHR^−/−^ mice. (D) Serum TG and T‐CHO levels of male HFD‐Flox and HFD‐LiGHR^−/−^ mice. (E) Serum LDL and HDL levels in male HFD‐Flox and HFD‐LiGHR^−/−^ mice. (F) Serum AST and ALT levels in male HFD‐Flox and HFD‐LiGHR^−/−^ mice. (G) Fasting blood glucose levels in HFD‐Flox and HFD‐LiGHR^−/−^ mice. (H) Glucose tolerance tests (GTT) in male HFD‐Flox and HFD‐LiGHR^−/−^ mice. (I) Insulin tolerance tests (ITT) in the indicated groups. (J) Representative images of PAS‐stained liver sections from HFD‐Flox and HFD‐LiGHR^−/−^ mice. Scale bar: 50 μm. (K) The α‐SMA and Col1a1 levels in liver tissues of HFD‐Flox and HFD‐LiGHR^−/−^ mice were detected using α‐SMA (1:2000) and Col1a1 (1:2000) antibodies. (L) Representative images of Masson's trichrome staining of liver sections from HFD‐Flox and HFD‐LiGHR^−/−^ mice. Scale bar: 100 μm. (M) Protein levels of IL6 and IL1β in liver tissues of HFD‐Flox and HFD‐LiGHR^−/−^ mice were detected.
**Figure S4:** Long‐term HFD accelerates age‐related hepatic pathologies in male LiGHR^−/−^ mice. (A) Schematic illustration of the experimental design. Male LiGHR^−/−^ mice of 3 months of age were fed a HFD continuously for 3 months and then switched to a regular diet until 12 months of age. Age‐matched LiGHR^−/−^ mice at 12 months of age were analyzed (*n* = 6 per group). (B) Representative Oil Red O staining of liver sections from LiGHR^−/−^ and HFD‐LiGHR^−/−^ mice. Scale bar: 100 μm. (C) Western blot analysis of senescence‐associated proteins p16, p21, and p53 in liver tissues from LiGHR^−/−^ and HFD‐LiGHR^−/−^ mice. (D) Western blot analysis of IL6 and TNFα in liver tissues from LiGHR^−/−^ and HFD‐LiGHR^−/−^ mice.
**Figure S5:** GHR knockdown promotes cellular senescence in FFA‐treated AML12 cells. (A) The *Ghr* mRNA levels were measured by RT‐qPCR. Three shRNA fragments were designed to assess GHR knockdown efficiency, *n* = 5. (B) The p21 and γH2AX protein levels in BSA‐control, BSA‐shGHR, FFA‐control, and FFA‐shGHR cells were measured using p21 (1:1000) and γH2AX (1:2000) antibodies, *n* = 4. (C) Cell proliferation of BSA‐control, BSA‐shGHR, FFA‐control, and FFA‐shGHR groups was assessed by CCK‐8 assay. (D) Representative images of Oil Red O and BODIPY staining of lipid droplets in cells. Scale bar: 50 μm. (E) Intracellular TG levels in cells from different groups. *n* = 6. (F) The mRNA expression of *Il6*, *Il1β*, and *Tnfα* in BSA‐control, BSA‐shGHR, FFA‐control, and FFA‐shGHR cells was compared, *n* = 3.
**Figure S6:** Hepatocyte‐specific GHR deficiency reduces lipid droplet accumulation in SubQ WAT and eWAT. (A) Representative BODIPY staining (green) of SubQ WAT and eWAT from 24M‐Flox and 24M‐LiGHR^−/−^ mice. Scale bar: 100 μm. (B) Representative images of SubQ WAT and eWAT from male HFD‐Flox and HFD‐LiGHR^−/−^ mice. Scale bar: 1 cm. (C) Weights of SubQ WAT and eWAT from HFD‐Flox and HFD‐LiGHR^−/−^ mice, *n* = 6. (D) Representative Oil Red O and H&E staining of SubQ WAT sections from HFD‐Flox and HFD‐LiGHR^−/−^ mice. (E) Representative Oil Red O and H&E staining of eWAT sections from HFD‐Flox and HFD‐LiGHR^−/−^ mice.
**Figure S7:** RNA‐seq analysis of liver tissues from male Flox and LiGHR^−/−^ mice. (A) FPKM values of hepatic *Igf1* expression in 24M‐Flox and 24M‐LiGHR^−/−^ mice (*n* = 4 per group). (B) RT‐qPCR analysis of *Igf1* mRNA expression in the livers of 24M‐Flox and 24M‐LiGHR^−/−^ mice. (C) FPKM values of hepatic *Igf1* expression in the livers of HFD‐Flox and HFD‐LiGHR^−/−^ mice (*n* = 4 per group). (D) RT‐qPCR analysis of *Igf1* mRNA expression in the livers of HFD‐Flox and HFD‐LiGHR^−/−^ mice. (E) PCA of liver RNA‐seq samples from 24M‐Flox and 24M‐LiGHR^−/−^ mice. (F) Volcano plot showing altered genes in liver tissues from 24M‐LiGHR^−/−^ mice compared to 24M‐Flox mice. (G) PCA of liver RNA‐seq samples from HFD‐Flox and HFD‐LiGHR^−/−^ mice. (H) Volcano plot showing altered genes in liver tissues from HFD‐LiGHR^−/−^ mice compared to HFD‐Flox mice. (I) Western blot analysis of AMPK and p‐AMPK protein levels in the liver of 24M‐Flox and 24M‐LiGHR^−/−^ mice (*n* = 6 per group). (J) Western blot analysis of mTOR and phosphorylated mTOR (p‐mTOR) protein levels in the livers of 24M‐Flox and 24M‐LiGHR^−/−^ mice (*n* = 6 per group). (K) Western blot analysis of p‐P65 and P65 protein levels in the livers of 24M‐Flox and 24M‐LiGHR^−/−^ mice (*n* = 6 per group). (L) Western blot analysis of SIRT5 and SIRT1 in the livers of 24M‐Flox and 24M‐LiGHR^−/−^ mice (*n* = 6 per group). (M) The expression of PPARγ, STAT5b, and p‐STAT5b in the livers of HFD‐Flox and HFD‐LiGHR^−/−^ mice was analyzed (*n* = 6 per group).
**Figure S8:** GHR deficiency suppresses p‐STAT5b‐PPARγ‐PDK4 signaling. (A) Venn diagram showing the overlap of down‐regulated genes in the liver of HFD‐fed LiGHR^−/−^ mice, down‐regulated genes in 24‐month‐old LiGHR^−/−^ mice, and PPARγ target genes from the ChEA and ChIP‐Atlas database. (B) FPKM values of AR, EP300, ESR1, FOXA2, and TEAD4 in liver tissues from 24M‐Flox and 24M‐LiGHR^−/−^ mice. (C) FPKM values of AR, EP300, ESR1, FOXA2, and TEAD4 in liver tissues from HFD‐Flox and HFD‐LiGHR^−/−^ mice. (D) Western blot analysis of STAT5b, PPARγ, and PDK4 protein levels in Mock and STAT5b‐OE cells (*n* = 3). (E) RT‐qPCR analysis of *Pparγ* mRNA expression in AML12 cells transfected with three independent siRNAs targeting PPARγ to evaluate knockdown efficiency (*n* = 3). (F) Western blot analysis of PPARγ and PDK4 in si‐Control and si‐PPARγ cells (*n* = 3). (G) RT‐qPCR analysis of *Pdk4* mRNA expression in si‐Control and si‐PPARγ cells. (H) Western blot analysis of CD36 and PDK4 protein expression in control and shGHR cells following FFA treatment for 48 h, with or without GH (500 ng/mL) for 1 h (*n* = 3).
**Figure S9:** GHR deficiency attenuates oxidative stress and activates autophagy in the liver of obese and aged mice. (A) ROS levels in liver tissues from HFD‐Flox and HFD‐LiGHR^−/−^ mice, measured by flow cytometry. (B) Antioxidant enzyme activities of SOD, CAT, and GSH‐Px in the liver tissues of 24‐month‐old Flox and LiGHR^−/−^ mice. (C) Western blot analysis of Beclin1, LC3B‐II/LC3B‐I ratio, and p62 protein levels in liver tissues from HFD‐Flox and HFD‐LiGHR^−/−^ mice. *n* = 6.
**Figure S10:** PDK4 inhibition alleviates age‐related hepatic pathology in aged Flox mice. (A) Schematic illustration of the experimental design. 20‐month‐old Flox mice were treated with vehicle or PDK4‐IN (10 mg/kg, i.p., once weekly) for 16 consecutive weeks (*n* = 6 per group). (B) Serum alanine aminotransferase (ALT) and aspartate aminotransferase (AST) levels in Flox mice treated with or without PDK4‐IN, as measured by ELISA. (C) Western blot analysis of senescence‐associated proteins p16, p21, and p53 in the livers of Flox mice treated with or without PDK4‐IN. (D) Western blot analysis of inflammatory markers IL6 and TNFα in the livers of Flox mice treated with or without PDK4‐IN.
**Table S1:** Detailed information of PCR primer sequences.
**Table S2:** Specific primers for RT‐qPCR.

## Data Availability

The data that support the findings of this study are available from the corresponding author upon reasonable request.

## References

[acel70741-bib-0001] Basu, R. , C. L. Boguszewski , and J. J. Kopchick . 2025. “Growth Hormone Action as a Target in Cancer: Significance, Mechanisms, and Possible Therapies.” Endocrine Reviews 46, no. 2: 224–280. 10.1210/endrev/bnae030. 39657053

[acel70741-bib-0002] Chang, A. Y. , V. F. Skirbekk , S. Tyrovolas , N. J. Kassebaum , and J. L. Dieleman . 2019. “Measuring Population Ageing: An Analysis of the Global Burden of Disease Study 2017.” Lancet Public Health 4, no. 3: e159–e167. 10.1016/s2468-2667(19)30019-2. PMC6472541 30851869

[acel70741-bib-0003] Coschigano, K. T. , A. N. Holland , M. E. Riders , E. O. List , A. Flyvbjerg , and J. J. Kopchick . 2003. “Deletion, but Not Antagonism, of the Mouse Growth Hormone Receptor Results in Severely Decreased Body Weights, Insulin, and Insulin‐Like Growth Factor I Levels and Increased Life Span.” Endocrinology 144, no. 9: 3799–3810. 10.1210/en.2003-0374. 12933651

[acel70741-bib-0004] Dabravolski, S. A. , E. E. Bezsonov , and A. N. Orekhov . 2021. “The Role of Mitochondria Dysfunction and Hepatic Senescence in NAFLD Development and Progression.” Biomedicine & Pharmacotherapy = Biomedecine & Pharmacotherapie 142: 112041. 10.1016/j.biopha.2021.112041. 34411916

[acel70741-bib-0005] Dominici, F. P. , G. Arostegui Diaz , A. Bartke , J. J. Kopchick , and D. Turyn . 2000. “Compensatory Alterations of Insulin Signal Transduction in Liver of Growth Hormone Receptor Knockout Mice.” Journal of Endocrinology 166, no. 3: 579–590. 10.1677/joe.0.1660579. 10974652

[acel70741-bib-0006] Dou, X. , Q. Fu , Q. Long , et al. 2023. “PDK4‐Dependent Hypercatabolism and Lactate Production of Senescent Cells Promotes Cancer Malignancy.” Nature Metabolism 5, no. 11: 1887–1910. 10.1038/s42255-023-00912-w. PMC10663165 37903887

[acel70741-bib-0007] Du, K. , D. S. Umbaugh , L. Wang , et al. 2025. “Targeting Senescent Hepatocytes for Treatment of Metabolic Dysfunction‐Associated Steatotic Liver Disease and Multi‐Organ Dysfunction.” Nature Communications 16, no. 1: 3038. 10.1038/s41467-025-57616-w. PMC11953480 40155379

[acel70741-bib-0008] Du, K. , L. Wang , J. H. Jun , et al. 2024. “Aging Promotes Metabolic Dysfunction‐Associated Steatotic Liver Disease by Inducing Ferroptotic Stress.” Nature Aging 4, no. 7: 949–968. 10.1038/s43587-024-00652-w. PMC12810195 38918603

[acel70741-bib-0009] Duan, J. , W. Dong , G. Wang , et al. 2023. “Senescence‐Associated 13‐HODE Production Promotes Age‐Related Liver Steatosis by Directly Inhibiting Catalase Activity.” Nature Communications 14, no. 1: 8151. 10.1038/s41467-023-44026-z. PMC10710422 38071367

[acel70741-bib-0010] Dujon, A. M. , K. Asselin , J. F. Lemaître , et al. 2026. “The Role of Selection for Function in Aging and Chronic Diseases: A Novel Evolutionary Perspective.” Aging Cell 25, no. 1: e70207. 10.1111/acel.70207. PMC12740078 40910442

[acel70741-bib-0011] Eguchi, T. , M. Abe , T. Tomita , et al. 2026. “Reversible Suppression of Autophagy in a Mouse Model Reveals Neuronal Resilience.” Science (New York, N.Y.) 392, no. 6805: 1363–1368. 10.1126/science.ady3911. 42348689

[acel70741-bib-0012] Fan, Y. , R. K. Menon , P. Cohen , et al. 2009. “Liver‐Specific Deletion of the Growth Hormone Receptor Reveals Essential Role of Growth Hormone Signaling in Hepatic Lipid Metabolism.” Journal of Biological Chemistry 284, no. 30: 19937–19944. 10.1074/jbc.M109.014308. PMC2740419 19460757

[acel70741-bib-0013] Gan, Q. , J. Huang , R. Zhou , et al. 2008. “PPAR{Gamma} Accelerates Cellular Senescence by Inducing p16INK4{Alpha} Expression in Human Diploid Fibroblasts.” Journal of Cell Science 121, no. Pt 13: 2235–2245. 10.1242/jcs.026633. 18544633

[acel70741-bib-0014] Gao, Y. , W. Zhang , L. Q. Zeng , et al. 2020. “Exercise and Dietary Intervention Ameliorate High‐Fat Diet‐Induced NAFLD and Liver Aging by Inducing Lipophagy.” Redox Biology 36: 101635. 10.1016/j.redox.2020.101635. PMC7365984 32863214

[acel70741-bib-0015] Guevara‐Aguirre, J. , A. Guevara , I. Palacios , M. Pérez , P. Prócel , and E. Terán . 2018. “GH and GHR Signaling in Human Disease.” Growth Hormone & IGF Research: Official Journal of the Growth Hormone Research Society and the International IGF Research Society 38: 34–38. 10.1016/j.ghir.2017.12.006. 29395968

[acel70741-bib-0016] Hwa, V. 2021. “Human Growth Disorders Associated With Impaired GH Action: Defects in STAT5B and JAK2.” Molecular and Cellular Endocrinology 519: 111063. 10.1016/j.mce.2020.111063. PMC7736371 33122102

[acel70741-bib-0017] Hwang, B. , N. H. Jeoung , and R. A. Harris . 2009. “Pyruvate Dehydrogenase Kinase Isoenzyme 4 (PDHK4) Deficiency Attenuates the Long‐Term Negative Effects of a High‐Saturated Fat Diet.” Biochemical Journal 423, no. 2: 243–252. 10.1042/bj20090390. 19627255

[acel70741-bib-0018] Jeon, J. H. , T. Thoudam , E. J. Choi , M. J. Kim , R. A. Harris , and I. K. Lee . 2021. “Loss of Metabolic Flexibility as a Result of Overexpression of Pyruvate Dehydrogenase Kinases in Muscle, Liver and the Immune System: Therapeutic Targets in Metabolic Diseases.” Journal of Diabetes Investigation 12, no. 1: 21–31. 10.1111/jdi.13345. PMC7779278 32628351

[acel70741-bib-0019] Kaltenecker, D. , M. Themanns , K. M. Mueller , et al. 2019. “Hepatic Growth Hormone—JAK2—STAT5 Signalling: Metabolic Function, Non‐Alcoholic Fatty Liver Disease and Hepatocellular Carcinoma Progression.” Cytokine 124: 154569. 10.1016/j.cyto.2018.10.010. 30389231

[acel70741-bib-0020] Kennedy, L. , V. Meadows , A. Sybenga , et al. 2021. “Mast Cells Promote Nonalcoholic Fatty Liver Disease Phenotypes and Microvesicular Steatosis in Mice Fed a Western Diet.” Hepatology (Baltimore, Md) 74, no. 1: 164–182. 10.1002/hep.31713. PMC9271361 33434322

[acel70741-bib-0021] Kim, Y. D. , Y. H. Kim , S. Tadi , et al. 2012. “Metformin Inhibits Growth Hormone‐Mediated Hepatic PDK4 Gene Expression Through Induction of Orphan Nuclear Receptor Small Heterodimer Partner.” Diabetes 61, no. 10: 2484–2494. 10.2337/db11-1665. PMC3447904 22698918

[acel70741-bib-0022] Kiourtis, C. , M. Terradas‐Terradas , L. M. Gee , et al. 2024. “Hepatocellular Senescence Induces Multi‐Organ Senescence and Dysfunction via TGFβ.” Nature Cell Biology 26, no. 12: 2075–2083. 10.1038/s41556-024-01543-3. PMC11628396 39537753

[acel70741-bib-0023] Kopchick, J. J. , D. E. Berryman , V. Puri , K. Y. Lee , and J. O. L. Jorgensen . 2020. “The Effects of Growth Hormone on Adipose Tissue: Old Observations, New Mechanisms.” Nature Reviews Endocrinology 16, no. 3: 135–146. 10.1038/s41574-019-0280-9. PMC7180987 31780780

[acel70741-bib-0024] Kundu, D. , L. Kennedy , T. Zhou , et al. 2023. “p16 INK4A Drives Nonalcoholic Fatty Liver Disease Phenotypes in High Fat Diet Fed Mice Through Biliary E2F1/FOXO1/IGF‐1 Signaling.” Hepatology (Baltimore, Md) 78, no. 1: 243–257. 10.1097/hep.0000000000000307. PMC10410572 36799449

[acel70741-bib-0025] Li, G. , Y. Hu , H. Zhao , et al. 2025. “Slow Metabolism‐Driven Amplification of Hepatic PPARγ Agonism Mediates Benzbromarone‐Induced Obesity‐Specific Liver Injury.” Advanced Science 12, no. 3: e2409126. 10.1002/advs.202409126. PMC11744575 39611414

[acel70741-bib-0026] List, E. O. , D. E. Berryman , J. Slyby , et al. 2022. “Disruption of Growth Hormone Receptor in Adipocytes Improves Insulin Sensitivity and Lifespan in Mice.” Endocrinology 163, no. 10: bqac129. 10.1210/endocr/bqac129. PMC9467438 35952979

[acel70741-bib-0027] Liu, H. , H. Zhang , H. Lou , et al. 2024. “ZEB1‐AS1 as a TRPML1 Inhibitor to Cause Lysosome Dysfunction and Cardiac Damage in Aged Mice.” Engineering 43: 183–200. 10.1016/j.eng.2024.09.020.

[acel70741-bib-0028] Liu, X. , Y. Gu , S. Kumar , et al. 2023. “Oxylipin‐PPARγ‐Initiated Adipocyte Senescence Propagates Secondary Senescence in the Bone Marrow.” Cell Metabolism 35, no. 4: 667–684.e6. 10.1016/j.cmet.2023.03.005. PMC10127143 37019080

[acel70741-bib-0029] Ma, R. , L. Ran , J. Geng , et al. 2026. “Adipose‐Specific GHR Knockout Confers Multidimensional Anti‐Aging Advantages via Adipose Tissue Remodelling and Enhanced Metabolic Elasticity.” Diabetes, Obesity & Metabolism 28, no. 6: 4741–4758. 10.1111/dom.70655. 41847844

[acel70741-bib-0030] Ma, W. Q. , X. J. Sun , Y. Zhu , and N. F. Liu . 2020. “PDK4 Promotes Vascular Calcification by Interfering With Autophagic Activity and Metabolic Reprogramming.” Cell Death & Disease 11, no. 11: 991. 10.1038/s41419-020-03162-w. PMC7673024 33203874

[acel70741-bib-0031] Ogrodnik, M. , S. Miwa , T. Tchkonia , et al. 2017. “Cellular Senescence Drives Age‐Dependent Hepatic Steatosis.” Nature Communications 8: 15691. 10.1038/ncomms15691. PMC5474745 28608850

[acel70741-bib-0032] Poudel, S. B. , R. R. Ruff , Z. He , et al. 2025. “The Impact of Inactivation of the GH/IGF Axis During Aging on Healthspan.” GeroScience 47, no. 3: 3027–3042. 10.1007/s11357-024-01426-3. PMC12181575 39535693

[acel70741-bib-0033] Ran, L. , X. Wang , A. Mi , et al. 2019. “Loss of Adipose Growth Hormone Receptor in Mice Enhances Local Fatty Acid Trapping and Impairs Brown Adipose Tissue Thermogenesis.” iScience 16: 106–121. 10.1016/j.isci.2019.05.020. PMC6545351 31154207

[acel70741-bib-0034] Shipley, J. M. , and D. J. Waxman . 2003. “Down‐Regulation of STAT5b Transcriptional Activity by Ligand‐Activated Peroxisome Proliferator‐Activated Receptor (PPAR) Alpha and PPARgamma.” Molecular Pharmacology 64, no. 2: 355–364. 10.1124/mol.64.2.355. 12869640

[acel70741-bib-0035] Sun, L. , L. Xu , T. Duan , et al. 2025. “CAV1 Exacerbates Renal Tubular Epithelial Cell Senescence by Suppressing CaMKK2/AMPK‐Mediated Autophagy.” Aging Cell 24, no. 5: e14501. 10.1111/acel.14501. PMC12073896 39887553

[acel70741-bib-0036] Thoudam, T. , D. Chanda , J. Y. Lee , et al. 2023. “Enhanced Ca(2+)‐Channeling Complex Formation at the ER‐Mitochondria Interface Underlies the Pathogenesis of Alcohol‐Associated Liver Disease.” Nature Communications 14, no. 1: 1703. 10.1038/s41467-023-37214-4. PMC10042999 36973273

[acel70741-bib-0037] Thoudam, T. , C. M. Ha , J. Leem , et al. 2019. “PDK4 Augments ER‐Mitochondria Contact to Dampen Skeletal Muscle Insulin Signaling During Obesity.” Diabetes 68, no. 3: 571–586. 10.2337/db18-0363. PMC6385748 30523025

[acel70741-bib-0038] Vázquez‐Borrego, M. C. , M. Del Río‐Moreno , M. Pyatkov , et al. 2023. “Direct and Systemic Actions of Growth Hormone Receptor (GHR)‐Signaling on Hepatic Glycolysis, De Novo Lipogenesis and Insulin Sensitivity, Associated With Steatosis.” Metabolism, Clinical and Experimental 144: 155589. 10.1016/j.metabol.2023.155589. PMC10843389 37182789

[acel70741-bib-0039] Vorhees, C. V. , and M. T. Williams . 2006. “Morris Water Maze: Procedures for Assessing Spatial and Related Forms of Learning and Memory.” Nature Protocols 1, no. 2: 848–858. 10.1038/nprot.2006.116. PMC2895266 17406317

[acel70741-bib-0040] Wakao, H. , R. Wakao , A. Oda , and H. Fujita . 2011. “Constitutively Active Stat5A and Stat5B Promote Adipogenesis.” Environmental Health and Preventive Medicine 16, no. 4: 247–252. 10.1007/s12199-010-0193-7. PMC3117211 21431790

[acel70741-bib-0041] Wan, J. , X. Wu , H. Chen , et al. 2020. “Aging‐Induced Aberrant RAGE/PPARα Axis Promotes Hepatic Steatosis via Dysfunctional Mitochondrial β Oxidation.” Aging Cell 19, no. 10: e13238. 10.1111/acel.13238. PMC7576254 32936538

[acel70741-bib-0042] Wang, X. , Z. Feng , S. Sun , et al. 2026. “cGAS‐STING‐Driven Inflammaging Cascade in Aging‐Related Retinal Diseases: From Pathological Mechanism to Therapeutic Potentials.” Ageing Research Reviews 114: 102983. 10.1016/j.arr.2025.102983. 41349895

[acel70741-bib-0043] Wu, Y. , C. Wang , H. Sun , D. LeRoith , and S. Yakar . 2009. “High‐Efficient FLPo Deleter Mice in C57BL/6J Background.” PLoS One 4, no. 11: e8054. 10.1371/journal.pone.0008054. PMC2777316 19956655

[acel70741-bib-0044] Yamauchi, S. , Y. Sugiura , J. Yamaguchi , et al. 2024. “Mitochondrial Fatty Acid Oxidation Drives Senescence.” Science Advances 10, no. 43: eado5887. 10.1126/sciadv.ado5887. PMC11506141 39454000

[acel70741-bib-0045] Yang, W. , D. M. Kim , W. Jiang , et al. 2023. “Suppression of FOXO1 Attenuates Inflamm‐Aging and Improves Liver Function During Aging.” Aging Cell 22, no. 10: e13968. 10.1111/acel.13968. PMC10577549 37602516

[acel70741-bib-0046] Young, J. A. , B. E. Henry , F. Benencia , et al. 2020. “GHR(−/−) Mice Are Protected From Obesity‐Related White Adipose Tissue Inflammation.” Journal of Neuroendocrinology 32, no. 11: e12854. 10.1111/jne.12854. PMC7554100 32350959

[acel70741-bib-0047] Yu, D. , X. Yang , X. Zhang , et al. 2026. “Deletion of Hepatic Growth Hormone Receptor Contributes to the Pathogenesis of Lean MAFLD by Elevating CD36.” Life Metabolism 5, no. 1: loaf037. 10.1093/lifemeta/loaf037. PMC12990309 41847434

[acel70741-bib-0048] Yu, L. , Q. Wan , Q. Liu , et al. 2024. “IgG Is an Aging Factor That Drives Adipose Tissue Fibrosis and Metabolic Decline.” Cell Metabolism 36, no. 4: 793–807.e5. 10.1016/j.cmet.2024.01.015. PMC11070064 38378001

[acel70741-bib-0049] Zeng, Q. , Y. Gong , N. Zhu , Y. Shi , C. Zhang , and L. Qin . 2024. “Lipids and Lipid Metabolism in Cellular Senescence: Emerging Targets for Age‐Related Diseases.” Ageing Research Reviews 97: 102294. 10.1016/j.arr.2024.102294. 38583577

[acel70741-bib-0050] Zhang, M. , Y. Zhao , Z. Li , and C. Wang . 2018. “Pyruvate Dehydrogenase Kinase 4 Mediates Lipogenesis and Contributes to the Pathogenesis of Nonalcoholic Steatohepatitis.” Biochemical and Biophysical Research Communications 495, no. 1: 582–586. 10.1016/j.bbrc.2017.11.054. 29128353

[acel70741-bib-0051] Zhang, X. , G. B. Xu , D. Zhou , and Y. X. Pan . 2018. “High‐Fat Diet Modifies Expression of Hepatic Cellular Senescence Gene p16(INK4a) Through Chromatin Modifications in Adult Male Rats.” Genes & Nutrition 13: 6. 10.1186/s12263-018-0595-5. PMC5853101 29564021

[acel70741-bib-0052] Zhang, Y. , R. Ma , J. He , et al. 2026. “Adipose‐Specific GHR Deletion Attenuates Brain Aging and Cognitive Decline in Aged Mice.” Aging Cell 25, no. 2: e70407. 10.1111/acel.70407. PMC12900898 41681058

