# Supplementary material for: Hepatocyte Growth Hormone Receptor Ablation Is Associated With Aging Phenotypes and Hepatic Mitochondrial Dysfunction
Source: Aging Cell. 2026 Sep 25;25(10):e70741. doi: 10.1111/acel.70741 (PMC13615307; doi:10.1111/acel.70741)
Supplement: Supplementary file 1 [file ACEL-25-e70741-s001.docx]

Supporting Information

**Hepatocyte Growth Hormone Receptor Ablation Is Associated with Aging Phenotypes and Hepatic Mitochondrial Dysfunction**

Kangkang Yang^1^, Yuli Jian^2^, Fusheng Pang^1^, Ming Ying^1^, Qian Yang^1^, Nian Liu^1^, Shujing Wang^2*^, Yingjie Wu^1,3*^

^1^Institute of Genome Engineered Animal Models for Human Diseases, National Center of Genetically Engineered Animal Models for International Research, Liaoning Provence Key Lab of Genome Engineered Animal Models, Dalian Medical University, Dalian, Liaoning, 116044, P. R. China

^2^Department of Biochemistry and Molecular Biology, College of Basic Medical Sciences, Liaoning Provincial Core Lab of Glycobiology and Glycoengineering, Dalian Medical University, Dalian, Liaoning, 116044, P. R. China

^3^Key Laboratory of Endocrine Glucose & Lipids Metabolism and Brain Aging, Ministry of Education, Shandong Provincial Hospital Affiliated to Shandong First Medical University & Shandong Academy of Medical Sciences, Jinan, Shandong, 250012, P. R. China

**^*^Corresponding Authors:**

Yingjie Wu, E-mail: yingjiewu@dmu.edu.cn

Shujing Wang, E-mail: wangshujing@dmu.edu.cn

**Supplemental Figures**

**Figure S1. Generation and characterization of hepatocyte-specific GHR knockout mice.**

(A) Schematic illustration of LiGHR^-/-^ mouse construction. (B) PCR genotyping of Flox and LiGHR^-/-^ mice. (C) PCR analyses of GHR expression in various tissues (liver, subcutaneous white adipose tissue (SubQ), epididymal white adipose tissue (Epi), perirenal white adipose tissue (Peri), mesenteric white adipose tissue (Mes), brown adipose tissue (BAT), heart, spleen, kidney, lung, brain, and small intestine). A truncated band resulting from Cre-mediated excision was detected exclusively in LiGHR^-/-^ liver tissue. (D) Survival curves for female Flox (n = 22) and LiGHR^-/-^ (n = 20) mice. (E) Glucose tolerance tests (GTT) in 24M male Flox and LiGHR^-/-^ mice, n = 6. (F) Insulin tolerance tests (ITT) in the indicated groups, n = 6.

**Figure S2. Hepatocyte-specific GHR ablation exacerbates hepatic histopathology in male LiGHR^-/-^ mice.**

(A) Representative SA-β-gal staining images of major metabolic tissues (brain, subcutaneous white adipose tissue (SubQ WAT), epididymal white adipose tissue (eWAT), kidney, and heart) from 24M-Flox and 24M-LiGHR^-/-^ mice. (B) Images of livers from 24M male Flox and LiGHR^-/-^ mice. Scale bar: 1 cm. (C) Liver weights of 24-month-old male Flox and LiGHR^-/-^ mice, n = 9. (D) Liver-to-body weight ratios of the indicated groups (n = 9 per group). (E) Immunohistochemical detection of γH2AX in the liver of 24M-Flox and 24M-LiGHR^-/-^ mice. Scale bar: 50 μm. (F) Immunofluorescence staining for α-SMA in liver tissues from 24M-Flox and 24M-LiGHR^-/-^ mice. Scale bar: 50 μm. (G) Western blot analysis of IL1β and IL6 protein levels in liver tissues of 24M-Flox and 24M-LiGHR^-/-^ mice. (H) Schematic illustration of the experimental design. Male LiGHR^-/-^ mice were fed regular chow and aged to 6, 12, or 24 months before sacrifice for subsequent analyses (n = 4 per group). (I) Representative Oil Red O staining images of liver sections from male LiGHR^-/-^ mice at 6, 12, or 24 months of age. Scale bar: 100 μm. (J) Western blot analysis of senescence-associated proteins p16 and p53 in liver tissues from male LiGHR^-/-^ mice at different ages. (K) Western blot analysis of inflammatory markers TNFα and IL6 in liver tissues from male LiGHR^-/-^ mice at different ages.

**Figure S3. Hepatocyte-specific GHR deficiency potentiates HFD-induced blood glucose disorder and hepatic injury.**

(A) Body weights of male HFD-Flox and HFD-LiGHR^-/-^ mice (n = 6 per group). (B) Representative images of H&E (Scale bar: 200 μm) and Oil Red O (Scale bar: 100 μm) staining of liver sections from HFD-Flox and HFD-LiGHR^-/-^ mice. (C) Liver-to-body weight ratios of HFD-Flox and HFD-LiGHR^-/-^ mice. (D) Serum TG and T-CHO levels of male HFD-Flox and HFD-LiGHR^-/-^ mice. (E) Serum LDL and HDL levels in male HFD-Flox and HFD-LiGHR^-/-^ mice. (F) Serum AST and ALT levels in male HFD-Flox and HFD-LiGHR^-/-^ mice. (G) Fasting blood glucose levels in HFD-Flox and HFD-LiGHR^-/-^ mice. (H) Glucose tolerance tests (GTT) in male HFD-Flox and HFD-LiGHR^-/-^ mice. (I) Insulin tolerance tests (ITT) in the indicated groups. (J) Representative images of PAS**-**stained liver sections from HFD-Flox and HFD-LiGHR^-/-^ mice. Scale bar: 50 μm. (K) The α-SMA and Col1a1 levels in liver tissues of HFD-Flox and HFD-LiGHR^-/-^ mice were detected using α-SMA (1:2000) and Col1a1 (1:2000) antibodies. (L) Representative images of Masson’s trichrome staining of liver sections from HFD-Flox and HFD-LiGHR^-/-^ mice. Scale bar: 100 μm. (M) Protein levels of IL6 and IL1β in liver tissues of HFD-Flox and HFD-LiGHR^-/-^ mice were detected.

**Figure S4. Long-term HFD accelerates age-related hepatic pathologies in male LiGHR^-/-^ mice.**

(A) Schematic illustration of the experimental design. Male LiGHR^-/-^ mice of 3 months of age were fed a HFD continuously for 3 months and then switched to a regular diet until 12 months of age. Age-matched LiGHR^-/-^ mice at 12 months of age were analyzed (n = 6 per group). (B) Representative Oil Red O staining of liver sections from LiGHR^-/-^ and HFD-LiGHR^-/-^ mice. Scale bar: 100 μm. (C) Western blot analysis of senescence-associated proteins p16, p21, and p53 in liver tissues from LiGHR^-/-^ and HFD-LiGHR^-/-^ mice. (D) Western blot analysis of IL6 and TNFα in liver tissues from LiGHR^-/-^ and HFD-LiGHR^-/-^ mice.

**Figure S5. GHR knockdown promotes cellular senescence in FFA-treated AML12 cells.**

(A) The *Ghr* mRNA levels were measured by RT-qPCR. Three shRNA fragments were designed to assess GHR knockdown efficiency, n = 5. (B) The p21 and γH2AX protein levels in BSA-control, BSA-shGHR, FFA-control, and FFA-shGHR cells were measured using p21 (1:1000) and γH2AX (1:2000) antibodies, n = 4. (C) Cell proliferation of BSA-control, BSA-shGHR, FFA-control, and FFA-shGHR groups was assessed by CCK-8 assay. (D) Representative images of Oil Red O and BODIPY staining of lipid droplets in cells. Scale bar: 50 μm. (E) Intracellular TG levels in cells from different groups. n = 6. (F) The mRNA expression of *Il6*, *Il1β*, and *Tnfα* in BSA-control, BSA-shGHR, FFA-control, and FFA-shGHR cells was compared, n = 3.

**Figure S6. Hepatocyte-specific GHR deficiency reduces lipid droplet accumulation in SubQ WAT and eWAT.**

(A) Representative BODIPY staining (green) of SubQ WAT and eWAT from 24M-Flox and 24M-LiGHR^-/-^ mice. Scale bar: 100 μm. (B) Representative images of SubQ WAT and eWAT from male HFD-Flox and HFD-LiGHR^-/-^ mice. Scale bar: 1 cm. (C) Weights of SubQ WAT and eWAT from HFD-Flox and HFD-LiGHR^-/-^ mice, n = 6. (D) Representative Oil Red O and H&E staining of SubQ WAT sections from HFD-Flox and HFD-LiGHR^-/-^ mice. (E) Representative Oil Red O and H&E staining of eWAT sections from HFD-Flox and HFD-LiGHR^-/-^ mice.

**Figure S7. RNA-seq analysis of liver tissues from male Flox and LiGHR^-/-^ mice.**

(A) FPKM values of hepatic *Igf1* expression in 24M-Flox and 24M-LiGHR^-/-^ mice (n = 4 per group). (B) RT-qPCR analysis of *Igf1* mRNA expression in the livers of 24M-Flox and 24M-LiGHR^-/-^ mice. (C) FPKM values of hepatic *Igf1* expression in the livers of HFD-Flox and HFD-LiGHR^-/-^ mice (n = 4 per group). (D) RT-qPCR analysis of *Igf1* mRNA expression in the livers of HFD-Flox and HFD-LiGHR^-/-^ mice. (E) PCA of liver RNA-seq samples from 24M-Flox and 24M-LiGHR^-/-^ mice. (F) Volcano plot showing altered genes in liver tissues from 24M-LiGHR^-/-^ mice compared to 24M-Flox mice. (G) PCA of liver RNA-seq samples from HFD-Flox and HFD-LiGHR^-/-^ mice. (H) Volcano plot showing altered genes in liver tissues from HFD-LiGHR^-/-^ mice compared to HFD-Flox mice. (I) Western blot analysis of AMPK and p-AMPK protein levels in the liver of 24M-Flox and 24M-LiGHR^-/-^ mice (n = 6 per group). (J) Western blot analysis of mTOR and phosphorylated mTOR (p-mTOR) protein levels in the livers of 24M-Flox and 24M-LiGHR^-/-^ mice (n = 6 per group). (K) Western blot analysis of p-P65 and P65 protein levels in the livers of 24M-Flox and 24M-LiGHR^-/-^ mice (n = 6 per group). (L) Western blot analysis of SIRT5 and SIRT1 in the livers of 24M-Flox and 24M-LiGHR^-/-^ mice (n = 6 per group). (M) The expression of PPARγ, STAT5b, and p-STAT5b in the livers of HFD-Flox and HFD-LiGHR^-/-^ mice was analyzed (n = 6 per group).

**Figure S8. GHR deficiency suppresses p-STAT5b-PPARγ-PDK4 signaling.**

(A) Venn diagram showing the overlap of down-regulated genes in the liver of HFD-fed LiGHR^-/-^ mice, down-regulated genes in 24-month-old LiGHR^-/-^ mice, and PPARγ target genes from the ChEA and ChIP-Atlas database. (B) FPKM values of AR, EP300, ESR1, FOXA2, and TEAD4 in liver tissues from 24M-Flox and 24M-LiGHR^-/-^ mice. (C) FPKM values of AR, EP300, ESR1, FOXA2, and TEAD4 in liver tissues from HFD-Flox and HFD-LiGHR^-/-^ mice. (D) Western blot analysis of STAT5b, PPARγ, and PDK4 protein levels in Mock and STAT5b-OE cells (n = 3). (E) RT-qPCR analysis of *Pparγ* mRNA expression in AML12 cells transfected with three independent siRNAs targeting PPARγ to evaluate knockdown efficiency (n = 3). (F) Western blot analysis of PPARγ and PDK4 in si-Control and si-PPARγ cells (n = 3). (G) RT-qPCR analysis of *Pdk4* mRNA expression in si-Control and si-PPARγ cells. (H) Western blot analysis of CD36 and PDK4 protein expression in control and shGHR cells following FFA treatment for 48 h, with or without GH (500 ng/mL) for 1 h (n = 3).

**Figure S9. GHR deficiency attenuates oxidative stress and activates autophagy in the liver of obese and aged mice.**

(A) ROS levels in liver tissues from HFD-Flox and HFD-LiGHR^-/-^ mice, measured by flow cytometry. (B) Antioxidant enzyme activities of SOD, CAT, and GSH-Px in the liver tissues of 24-month-old Flox and LiGHR^-/-^ mice. (C) Western blot analysis of Beclin1, LC3B-II/LC3B-I ratio, and p62 protein levels in liver tissues from HFD-Flox and HFD-LiGHR^-/-^ mice. n = 6.

**Figure S10. PDK4 inhibition alleviates age-related hepatic pathology in aged Flox mice.**

(A) Schematic illustration of the experimental design. 20-month-old Flox mice were treated with vehicle or PDK4-IN (10 mg/kg, i.p., once weekly) for 16 consecutive weeks (n = 6 per group). (B) Serum alanine aminotransferase (ALT) and aspartate aminotransferase (AST) levels in Flox mice treated with or without PDK4-IN, as measured by ELISA. (C) Western blot analysis of senescence-associated proteins p16, p21, and p53 in the livers of Flox mice treated with or without PDK4-IN. (D) Western blot analysis of inflammatory markers IL-6 and TNF-α in the livers of Flox mice treated with or without PDK4-IN.

**Supplemental Tables**

**Table S1: Detailed information of PCR primer sequences.**

| **Gene** | **Forward (5′→3′)** | **Reverse (5′→3′)** |
| --- | --- | --- |
| GHR | CATTCTTTTCTGGGATGCTAT | CGGACATTGCATCTGTGATT |
| Albumin-Cre | TAGGAACCAATGAAATGCGAGGT | AACCAGCGTTTTCGTTCTGC |

**Table S2: Specific primers for** **RT-qPCR.**

| **Gene** | **Forward (5′→3′)** | **Reverse (5′→3′)** |
| --- | --- | --- |
| *Ghr* | TCCCAGACTACACCACGGTTCAC | GTCAGGCAAAGGCAAAGCAGTTG |
| *Cdkn2a* | CCCAACGCCCCGAACT | GCAGAAGAGCTGCTACGTGAA |
| *Cdkn1a* | GCAGATCCACAGCGATATCC | CAACTGCTCACTGTCCACGG |
| *Cd36* | ATGGGCTGTGATCGGAACTG | GTCTTCCCAATAAGCATGTCTCC |
| *Fatp1* | CTGGGACTTCCGTGGACCT | TCTTGCAGACGATACGCAGAA |
| *Fatp2* | CGAGACGAGACGCTCACCTA | ACGAATGTTGTAGTTGAGGCAC |
| *Fatp3* | ATGGGGCTCCACCTATGGG | TGGCACTGTAGAACCTTCAGA |
| *Fatp4* | ACTGTTCTCCAAGCTAGTGCT | GATGAAGACCCGGATGAAACG |
| *Fatp5* | TCTATGGCCTAAAGTTCAGGCG | CTTGCCGCTCTAAAGCATCC |
| *Fatp6* | GTCATGGCTCACAGGATTGGG | CACGTCCTCGTAGGTGTAGAC |
| *Pparg* | GGAAGACCACTCGCATTCCTT | GTAATCAGCAACCATTGGGTCA |
| *Pdk4* | AGGGAGGTCGAGCTGTTCTC | GGAGTGTTCACTAAGCGGTCA |
| *Il6* | CTTCCATCCAGTTGCCTTCTTG | AATTAAGCCTCCGACTTGTGAAG |
| *Il1β* | TTGAAGAAGAGCCCATCCTC | GTCTAATGGGAACGTCACAC |
| *Tnfα* | CCAAAGGGATGAGAAGTTCC | CTTGGTGGTTTGCTACGAC |
| *Igf1* | GGATGAGTGTTGCTTCCGGA | TAGGGACGGGGACTTCTGAG |
| β-Actin | GGCTGTATTCCCCTCCATCG | CCAGTTGGTAACAATGCCATGT |
